# GPR65 is a novel immune biomarker and regulates the immune microenvironment in lung adenocarcinoma

**DOI:** 10.3389/fimmu.2025.1572757

**Published:** 2025-05-30

**Authors:** Hanxu Zhou, Zhi Chen, Shuang Gao, Chaoqun Lian, Junjie Hu, Jin Lu, Lei Zhang

**Affiliations:** ^1^ Department of General Surgery, Second Affiliated Hospital of Bengbu Medical University, Graduate Institute of Bengbu Medical University, Bengbu Medical University, Bengbu, Anhui, China; ^2^ Department of Medical Oncology, The First Affiliated Hospital of USTC, Division of Life Sciences and Medicine, University of Science and Technology of China, Hefei, Anhui, China; ^3^ Department of Biochemistry and Molecular Biology, School of Laboratory Medicine, Bengbu Medical University, Bengbu, Anhui, China; ^4^ Department of Radiotherapy, the Second Affiliated Hospital of Bengbu Medical University, Bengbu, Anhui, China; ^5^ Department of Human Anatomy, Key Laboratory of Computational Medicine and Intelligent Health of Anhui Higher Education Institutes, Bengbu Medical University, Bengbu, Anhui, China; ^6^ Department of General Surgery, The Second Affiliated Hospital of Bengbu Medical University, Bengbu, Anhui, China

**Keywords:** Gpr65, biomarker, prognosis, lung adenocarcinoma, immune microenvironment

## Abstract

**Background:**

The tumor microenvironment (TME) plays a crucial role in the progression of lung adenocarcinoma (LUAD), and it may serve as the best prognostic predictor of LUAD. GPR65 is an extracellular pH-sensing G protein-coupled receptor and a glycosphingolipid receptor, which is engaged in the functions of regulating tumor immunity. However, the prognostic value of GPR65 and its relevance to immune infiltration in LUAD are unknown.

**Methods:**

The proportion of immune, stromal and tumor cells in LUAD samples was assessed by ESTIMATE algorithm scores with RNA sequence data and clinical information from LUAD patients downloaded from The Cancer Genome Atlas (TCGA) database. We screened differential genes (DEGs) in the immune and stromal components, and then screened modular genes by the WGCNA algorithm, which were intersected with DEGs and incorporated into the LASSO-COX regression model. Additionally, nomogram containing GPR65 and clinical features were constructed for predicting patient prognosis. Then, the correlation between GPR65 and immune cell infiltration was assessed by CIBERSORT, and the impact of hub gene on immunotherapy was determined using correlation analysis between GPR65 and immune checkpoint molecules. Finally, we confirmed the expression of GPR65 in LUAD by Western Blot, Quantitative Real-time PCR and Immunohistochemistry.

**Results:**

In our study, we found that low expression of GPR65 was associated with poorer overall survival and primary treatment outcome in patients with LUAD. Moreover, GPR65 expression was found to be closely correlated with multiple tumor infiltrating immune cells (TIICs) and immune checkpoint molecules. Immunohistochemistry and Quantitative Real-time PCR results confirmed that the transcription levels and protein expression levels of GPR65 in LUAD tissues were significantly lower than in normal tissues. Western Blot results showed that the expression of GPR65 in human normal lung epithelial cell lines was significantly higher than the expression level in LUAD cell lines.

**Conclusions:**

GPR65 may be an important immune biomarker in the prognosis and diagnosis of LUAD.

## 1 Introduction

Lung cancer is the most common cancer worldwide and the leading cause of cancer-related death (1). More than 1.8 million people are diagnosed with lung cancer each year, and about 1.6 million die of it (2). Non-small cell lung cancer (NSCLC) accounts for approximately 85% of new lung cancer cases (3). In fact, lung adenocarcinoma (LUAD) has become the most common pathological subtype of NSCLC (4). Although surgery has achieved satisfactory performance in the treatment of early-stage lung cancer, some of the current treatments are still difficult to improve the 5-year survival rate of lung cancer patients with recurrence, metastasis and first diagnosed stage IV (5). In addition, the mechanisms involved in the prognosis of LUAD patients remain unclear, and the heterogeneity of tumors makes it difficult to accurately assess the survival prognosis of each patient (6–8). Therefore, there is an urgent need to accurately and individually assess and improve the survival rate of LUAD patients.

Tumor progression is typically considered a multistep process involving only genetic and epigenetic variation in tumor cells (9). However, many studies have shown that the composition of tumor microenvironment (TME) and the proportion of stromal cells differ between different tumors, meaning that the microenvironment on which tumor cells depend for growth and survival plays a crucial role in tumor progression (10, 11). TME usually includes immune cells, stromal cells, extracellular matrix (ECM), other secretory molecules and blood and lymphovascular networks that intertwine and communicate with each other and the heterogeneous cancer cells (12, 13). Novel evidence suggested that tumor infiltrating immune cells (TIICs) are important indicators in indicating tumor spread, recurrence, metastasis and response to immunotherapy (14, 15). A study reported heterogeneity of EGFR mutations in early LUAD, involving complex interactions between tumor cells, stromal cells and immune cell infiltration in TME (16). Although immunotherapy has shown significant clinical benefit in many cancers, its benefits are limited to a small subset of patients (17). A comprehensive analysis of the various components and pathways of LUAD in TME, identification of promising biomarkers and prediction of their response to immune checkpoint inhibitors (ICIs) are key steps in improving the effectiveness of immunotherapy and developing novel immunotherapeutic strategies, and will further contribute to the search for new therapeutic targets.

GPR65 (also known as T-cell death-associated gene 8, TDAG8) is an extracellular pH-sensing G protein-coupled receptor involved in the regulation of cancer cell metastasis and proliferation, immune cell function, inflammation and angiogenesis, etc. (18–20). The human GPR65 gene has been localized to chromosome 14q31-32.1, a position found to be associated with T-cell lymphoma and leukemia-related abnormalities, etc. In the immune system, GPR65 is normally expressed in lymphocytes, leukocytes and macrophages (21–23).

Current studies have not elucidated the relationship between GPR65 and LUAD immunity, so here, this study aimed to identify a reliable LUAD biomarker for predicting patient prognosis and treatment response. We studied the transcriptomic data and screened the potential key gene of LUAD —GPR65 by bioinformatics analysis in The Cancer Genome Atlas (TCGA) database. Next, we analyzed the correlation between GPR65 high and low risk groups and clinicopathological features and TIIC infiltration. Then, we studied the correlation between the expression of GPR65 and immune checkpoint molecules using the TCIA database.

In addition, we performed experimental validation by Western Blot, Quantitative Real-time PCR and Immunohistochemistry, and the final results confirmed that GPR65 is a valuable prognostic biomarker and may be a promising therapeutic target for LUAD.

## 2 Materials and methods

### 2.1 Collection and processing of gene matrix data

Transcriptomic data from 594 LUAD patients (535 tumour samples and 59 normal samples) and clinical data from 500 cases with survival time >30 days were collected from the TCGA dataset (https://cancergenome.nih.gov/) and finally a total of 490 LUAD patients with clinical information were obtained. The study was approved by the Ethics Committee of the Second Affiliated Hospital of Bengbu Medical College with reference number 109, in accordance with the Declaration of Helsinki, and used data available in the TCGA public database in accordance with the TCGA policy. The datasets involved in this study can all be found in online databases, which are all freely available to the public.

### 2.2 Analyze and identify DEGs in tumor microenvironment

The ESTIMATE algorithm was used to assess the composition of the TME. The results were expressed as four scores, ESTIMATEScore, ImmuneScore, StromalScore and TumorPurity, corresponding to the proportion of immune cells plus stromal cells, immune cells, stromal cells and tumor cells, respectively. All genes of the tumor samples were sorted by their expression levels and screened for DEGs using the “limma” package of R. DEGs were identified based on the following criteria as follows: |log2 fold change (FC)| > 1 and adjusted *p*-value < 0.05. In addition, the “VennDiagram” package was used to screen genes with similar expression levels in stromal cells and immune cells.

### 2.3 Survival analysis and LASSO regression

The clinicopathological characteristics of each sample were evaluated using the Wilcoxon rank sum and Kruskal-Wallis rank sum tests, which also helped to explain the relationship between results and clinical stages. To divide the sample into high and low subgroups, survival analysis using the median was conducted. The difference of *p* < 0.05 was considered significant using the R package “survival” and “ survivor “. LASSO regressions were performed using the glmnet R package using 10-fold cross-validation, with the penalty parameter (lambda) set to lambda.1se (the lambda that corresponds to the most plausible model within one standard error of the smallest error) to ensure that the for optimal performance of the prognostic model.

### 2.4 GO and KEGG function enrichment analysis of TME-associated DEGs

The Kyoto Encyclopedia of Genes and Genomes (KEGG) and Gene Ontology (GO) were used for TME-related analyses, revealing their functions in cellular components (CC), biological processes (BP), molecular functions (MF) and showing pathway enrichment results. The “ggplot2”, “enrichment map” and “cluster analyser” packages in R were used for GO and KEGG analysis. *p* < 0.05 and q < 0.05 were statistically significant.

### 2.5 Weighted gene co-expression network analysis

TCGA gene expression files of 594 LUAD samples were used to construct a scale-free correlation network using the R package “WGCNA” to construct the scale-free network. Here, we set the minimum module size to 20, cut height to 0.25 and performed screening to identify candidate module genes for the next analysis.

### 2.6 Relevance of GPR65 expression to clinicopathological features

Differences in overall survival (OS) and progression-free survival (PFS) between the low and high GPR65 expression groups were shown using Kaplan-Meier survival analysis. R language with the “pheatmap” package was used to create heat maps of single genes with clinical information. The GPR65 expression levels were then associated with clinical features using univariate and multivariate Cox regression analysis models.

### 2.7 Assessment of the detection efficacy of GPR65 on the prognostic impact of LUAD

The stepAIC algorithm run in the R “rms” package was used to construct an optimal prognostic model. C-index curves and correction curves were plotted in the R package “pec” and “regplot”, respectively to test the prognostic accuracy of GPR65 in predicting LUAD.

### 2.8 Deeper analysis of the relationship between GPR65 and tumor immune microenvironment and immunotherapy

To explore the role of GPR65 in the TME of LUAD, we utilized gene set enrichment analysis (GSEA) using GSEA version 3.0 (Broad Institute, Cambridge, MA, United States) to validate the results of KEGG and HALLMARK dataset enrichment analysis. Differences were considered significant if the NOM *p*-value < 0.05 and the FDR < 0.25. In addition, to determine the relative abundance of TIICs in LUAD samples, we estimated the extent of infiltration using the CIBERSORT algorithm. Samples with *p* < 0.05 had significant differences in immune cell infiltration between the two groups. In addition, we performed a correlation analysis between the expression of GPR65 in TME and immune cell infiltration. Moreover, the correlation between the expression levels of LUAD and immune checkpoint molecules in LUAD was identified by the R package “ggExtra” and “ggpubr”. *p* < 0.05 was set as statistically significant. The role of GPR65 in immunotherapy was further analyzed using the data of PD1 and CTLA4 treatment in LUAD patients from the TCIA website (https://tcia.at/home).

### 2.9 Cell culture

Human LUAD cell lines (A549 and H1299) and human lung epithelial cells (2B) were provided by the Department of Biochemistry and Molecular Biology Laboratory, Research Center, Bengbu Medical College (purchased from The Cell Bank of the Chinese Academy of Sciences, Shanghai, China) and cultured in RPMI1640 medium containing 10% fetal bovine serum and DMEM (High Glucose) medium (Gibco, Thermo Fisher, Beijing, China). The cells were then incubated at 37°C in a 5% CO2 incubator.

### 2.10 Clinical specimens and immunohistochemistry

Primary cancer tissues and adjacent paracancerous tissues were collected from lung adenocarcinoma patients at the First Affiliated Hospital of Bengbu Medical College, and processed by formalin fixation and paraffin embedding. Paraffin blocks of human LUAD tissue and paracancerous tissue were sectioned, then dewaxed, hydrated, hot repaired, sealed, and added antibodies (primary antibodies: rabbit polyclonal anti-GPR65 antibody, 1:200, Bioss, China, bs-7668R; secondary antibodies: HRP-labeled goat anti-rabbit, 1: 200, Servicebio, China, GB23303); DAB kit was stained, dehydrated, transparent, sealed, and observed under a microscope. GPR65 expression levels were determined by assessing the intensity of cell staining. The negative control group does not use the primary antibody to exclude the impact of non-specific secondary antibody binding on the experiment. The immunohistochemical results were interpreted by two pathologists who read the slices doubleblindly. And four visual fields were randomly collected in each case.

### 2.11 Western blot

Cells were lysed in RIPA lysate (Biosharp, China) to obtain protein lysates. Protein was loaded onto SDS-PAGE gel (Epizyme, USA) and transferred to a PVDF membrane (Biosharp, China). Then, 5% powdered skim milk was closed for 1 h at room temperature, membranes were washed thrice with TBST for 10 min each time. The following antibodies were incubated with the membranes at 4°C overnight: rabbit anti-GPR65 antibody (1:2000, bs-7668R, Bioss, China), and actin (1:1000, GB11001, Servicebio, China). The membranes were then incubated with HRP-labeled anti-rabbit secondary antibody (1:5000) under ambient temperature for 60 min. The membranes were washed thrice with TBST for 600 s each time. Protein bands were displayed using a fully automatic chemiluminescence imager. Finally, the results were analyzed using imageJ software. All target band results were normalized against the internal reference before analysis by using GAPDH as the internal reference.

### 2.12 Quantitative real-time PCR

Total RNA was extracted from tissues using TRIzol reagent (Solarbio). cDNA reverse transcription was performed using HiScript^®^ kit (Vazyme) for the purpose of quantifying prognostic gene levels. The expression levels of the prognostic genes were measured using SYBR qPCR Master Mix (Vazyme). The primer sequences used in this experiment were as follows: GPR65, 5’-GCATTGCCGTTGATCGGTATT-3’ (Forward Primer) and 5’-CGTCCTGAACAAGTTGAGGTT-3’ (Reverse Primer) and GAPDH, 5’-GGAGCGAGATCCCTCCAAAAT-3’ (Forward Primer) and 5’-GGCTGTTGTCATACTTCTCATGG-3’ (Reverse Primer). GPR65 used GAPDH as an internal reference.

### 2.13 Colony formation assay

H1299 or A549 cells, commonly used cell lines in lung cancer research, were carefully seeded in 6-well plates at a density of 1000 cells per well. This seeding density was chosen to ensure optimal cell growth and colony formation. The plates were then placed in a controlled incubator for a period of 14 days, allowing ample time for the cells to proliferate and form visible colonies. After the completion of clonal culture, use a camera to take photographs, count and statistically analyze the number of clones for data analysis.

### 2.14 Transwell assay

Transwell assays were performed using the HTS transwell-24 system (Corning, NY, USA), which consists of 24 Boyden chambers with transwell membranes having a pore size of 8 μm. The migration assays were carried out following the established protocol as described in a previous publication. In the invasion assays, the upper chambers were pre-coated with matrigel to mimic the extracellular matrix environment. Subsequently, cells that migrated to the lower surface were fixed, stained, and quantified after 8-12 hours of seeding.

### 2.15 Statistical analysis

Statistical analyses were conducted using R software (version 4.2.1; R Foundation for Statistical Computing, Vienna, Austria; https://www.r-project.org/) and GraphPad Prism software (version 8.0; GraphPad Software, San Diego, CA, USA). Prior to applying parametric tests, relevant assumptions were evaluated. For instance, normality of data or residuals was assessed using methods such as the Shapiro-Wilk test, and homogeneity of variances was checked using Levene’s test where appropriate. For comparisons between paired samples, such as carcinoma and adjacent normal tissues from the same patient in immunohistochemistry analyses, the paired-samples t-test was employed, contingent upon the approximate normality of the paired differences. For comparisons of means across more than two independent groups (e.g., expression levels between human LUAD cell lines and human lung epithelial cells, or analysis of qRT-PCR results across conditions), one-way Analysis of Variance (ANOVA) was used, provided its assumptions of normality and homogeneity of variance were reasonably met. The Spearman rank correlation test was utilized to determine the monotonic association between variables, particularly when the assumption of bivariate normality for Pearson correlation was not satisfied or when assessing rank-based relationships. Unless otherwise specified, results were expressed as average value ± standard deviation (SD). All statistical tests were two-tailed, and p-values < 0.05 were considered statistically significant. Significance levels are indicated in figures and tables as follows: * p < 0.05, ** p < 0.01, and *** p < 0.001.”

## 3 Results

### 3.1 The workflow chart of this study

First, we downloaded the transcriptome RNA sequence data of 594 LUAD patients from the TCGA database. The ESTIMATE algorithm was first used for calculation, followed by the WGCNA algorithm to identify candidate module genes and take the intersection with TIME-DEGs. Then the LASSO-Cox regression model was used to finally obtain the four best genes, and we focused on GPR65 for a subsequent series of analyses, including survival and clinicopathological feature correlation analysis, cox regression, GSEA and correlation with TIICs and ICs.

### 3.2 Correlation between ESTIMATEScore and TumorPurity and survival of patients with LUAD

**Figure 1:**
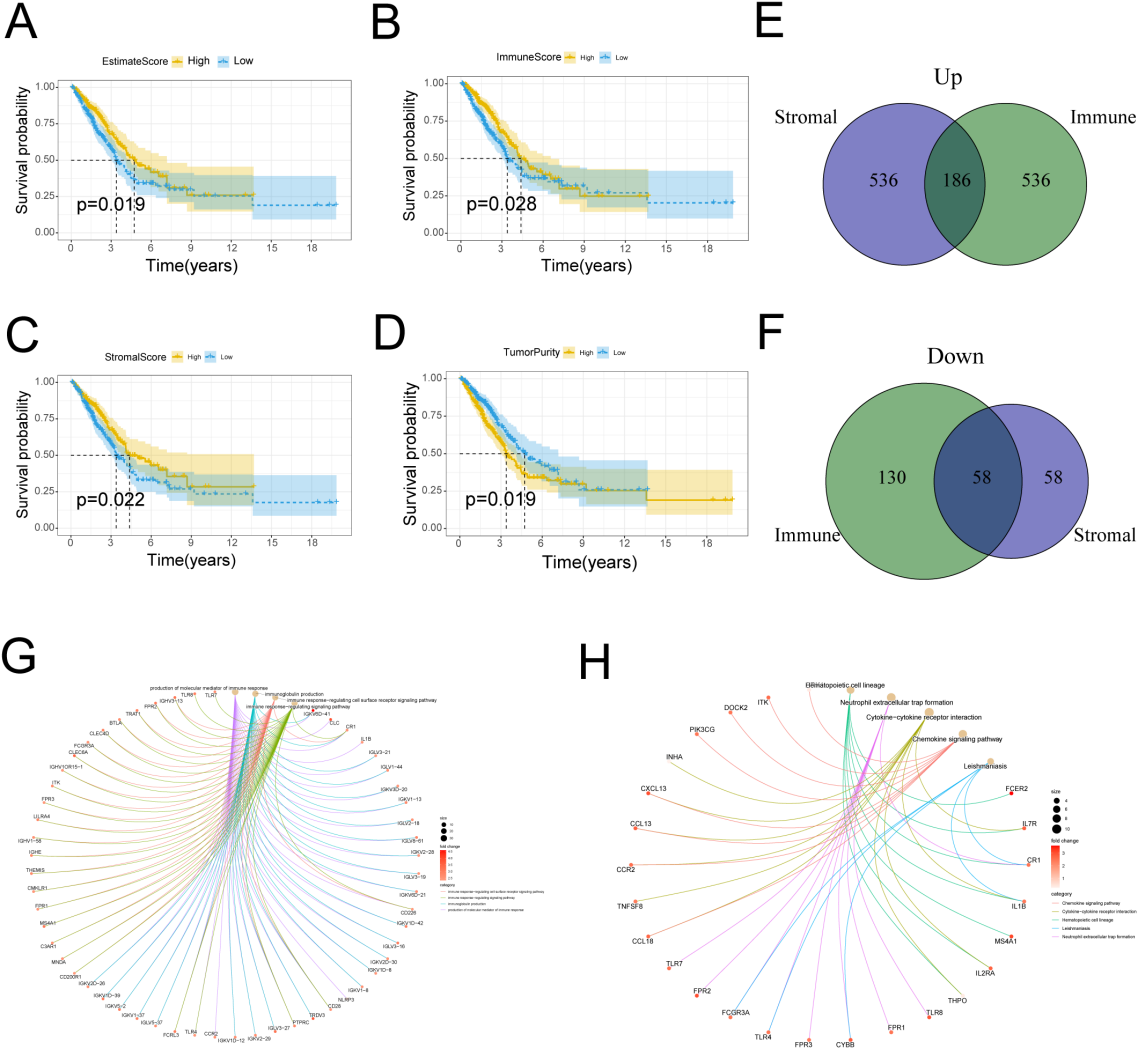
Correlation of scores and tumor purity with the survival of patients with LUAD. **(A)** Kaplan–Meier survival analysis for LUAD patients grouped into high or low score ESTIMATEScore determined by the comparison with the median (P = 0.019 by log-rank test). **(B)** Kaplan–Meier survival curve for ImmuneScore with P = 0.028 by log-rank test. **(C)** Survival analysis with Kaplan–Meier method for LUAD patients grouped by StromalScore (P = 0.022 by log-rank test). **(D)** Kaplan–Meier survival analysis for TumorPurity with *P* = 0.019 by log-rank test. **(E)** Venn plot of commonly upregulated DEGs in the stromal and immune components (|log2 fold change (FC)|>1, adj P-value < 0.05). **(F)** Venn plot showing common down-regulated DEGs shared by ImmuneScore and StromalScore. (|log2 fold change (FC)|>1, adj P-value < 0.05). **(G-H)** Gene Ontology (GO) and Kyoto Encyclopedia of Genes and Genomes (KEGG) enrichment analysis of differentially expressed genes (DEGs).

In this study, we first compared survival benefit for ESTIMATEScore, ImmuneScore, StromalScore and TumorPurity. Using the median as the cutoff value, patients were divided into high and low subgroups, and Kaplan-Meier curves were plotted. Our study showed that ESTIMATEScore, ImmuneScore and StromalScore were all positively correlated with OS (**Figures 1A–C**; *p* = 0.019, *p* = 0.028 and *p* = 0.022, respectively, log-rank test). However, TumorPurity was negatively associated with survival probability in LUAD patients (**Figure 1D**; *p* = 0.019, log-rank test). These results indicate that both TumorPurity and the immune and stromal components of TME are significantly related to the prognosis of LUAD patients.

### 3.3 Association between immune microenvironment scores and clinicopathological features in patients with LUAD

To elucidate the association of the three immune microenvironment scores with the clinicopathology in LUAD, we sequentially appraised age, anatomic site, gender and stage, respectively. The results showed that patients older than 65 years, female and stage I patients, they achieved higher ImmuneStore, while there was no difference in anatomic site (**Supplementary Figures 1A–D**, *p* < 0.05). Moreover, StromalStore also maintained the tendency of age and gender, but there was no difference in Stage (**Supplementary Figure 1E–H**, *p* < 0.05). In addition, ESTIMATEStore also maintained the same track with ImmuneStore (**Supplementary Figure 1I–L**, *p* < 0.05). The above results demonstrated that the components of the immune microenvironment in LUAD patients altered with age, gender differences and tumor progression in the organism.

### 3.4 DEGs for stromal and immune scoring demonstrate the corresponding immune functions and immune pathways

To elicit variation in transcriptomic gene expression across the different ImmuneStore and StromalStore, a total of 910 DEGs were obtained in the ImmuneScore with |log_2_FC| >1.5 and adjusted *p*-value < 0.05, including 722 up-regulated genes and 188 down-regulated genes. In the StromalScore, 838 DEGs were incorporated, with 722 up-regulated genes and 116 down-regulated genes. Venn diagram showed 186 up-regulated genes and 58 down-regulated genes in both of the above scores, and these 244 DEGs can be called TME-related DEGs (**Figures 1E–F**). These genes were further inputted into GO and KEGG for analysis. **Figure 1G** showed the pathways significantly enriched by GO, with gene sets mainly involved in immune response-related functions, such as the production of molecular mediator of immune response and immunoglobulin production. The results of KEGG analysis indicated that DEGs are involved in certain immune-related functions, including cytokine-cytokine receptor interaction and neutrophil extracellular traps formation (**Figure 1H**).

### 3.5 Immune microenvironment and WGCNA identified GPR65 as a hub gene for LUAD

**Figure 2:**
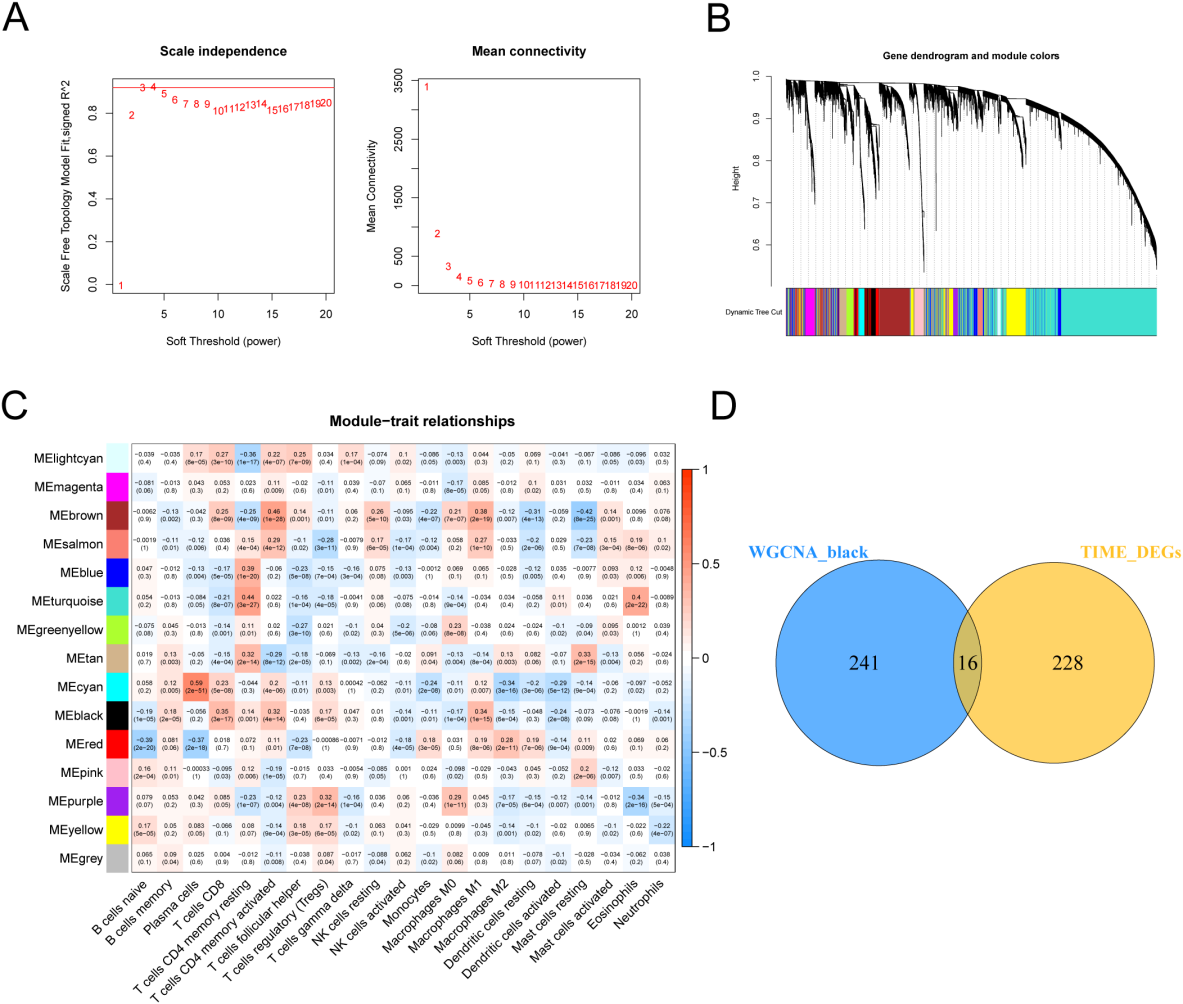
WGCNA and DEGs identified GPR65 as a hub gene for LUAD. **(A)** Analysis of network topology for various soft-threshold powers. **(B)** Clustering dendrograms of genes, with dissimilarity based on topological overlap, together with assigned module colors. **(C)** Analysis of module-trait relationships of LUAD based on TCGA data. **(D)** Venn diagram of WGCNA and DEGs.

**Figure 3:**
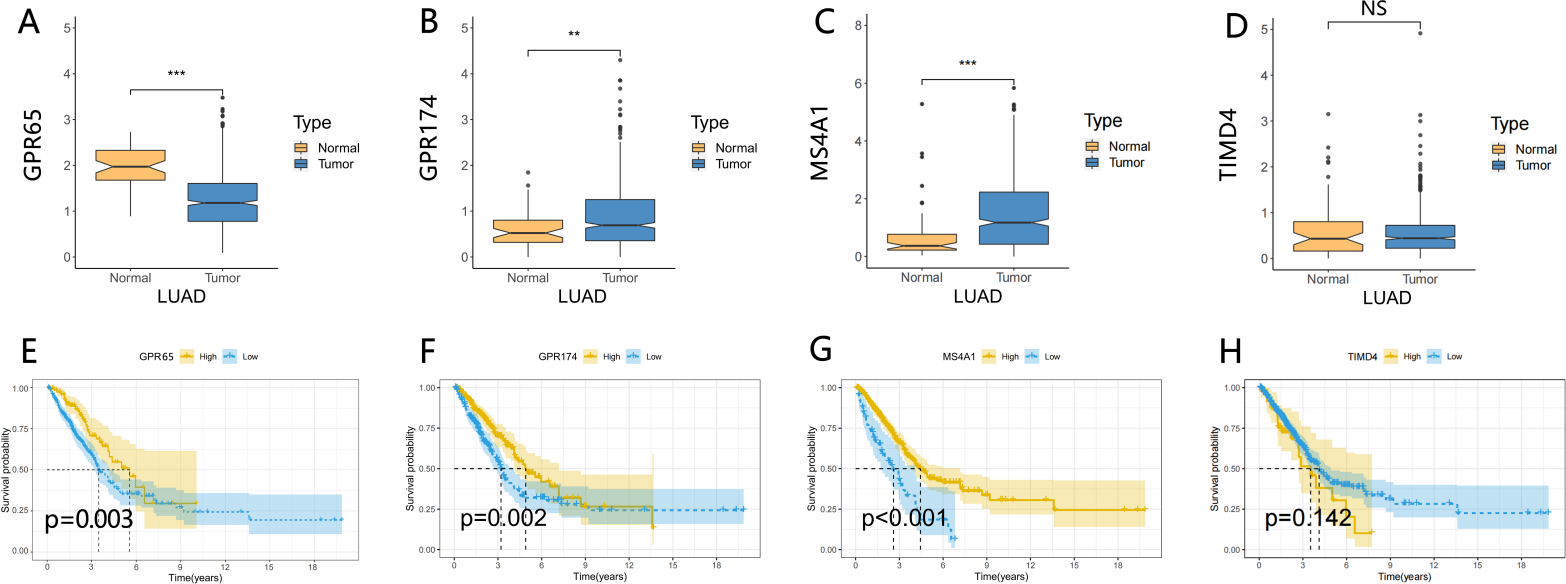
Integrated analysis of GPR65、GPR174、MS4A1 and TIMD4. **(A–D)** Different expression of GPR65、GPR174、MS4A1 and TIMD4 in the normal and cancer tissue; **(E–H)** Kaplan-Meier survival analysis between 4 gene expression levels and prognosis in LUAD patients.

Our previous study evaluated the TME of LUAD by using the ESTIMATE algorithm. To further identify the cellular components and forms of interaction in the LUAD microenvironment, we first analyzed the infiltration abundance of 21 immune cell types in LUAD using the CIBERSORT algorithm. Next, we determined a soft threshold of 4 by the WGCNA analysis method (**Figure 2A**). By merging the modules with the closest interactions (**Figure 2B**), we finally selected the most important cell population of anti-tumor cells, CD8+ T cells. We found that the black module was most closely related to CD8+ T cells (**Figure 2C**). By extracting the genes in the module with connectivity greater than 0.3, a Venn diagram of black module genes with TIME differential genes was drawn, resulting in a total of 16 differential genes (**Figure 2D**). Then, these genes were input into the LASSO-Cox regression model (**Supplementary Figure 2A**), and the best performance could be obtained by the combination of 4 genes (GPR65, GPR174, MS4A1, TIMD4) (**Supplementary Figure 2B**). Next, Wilcoxon rank sum test was performed for each of these 4 genes, and the results showed that the expression of GPR65 was significantly lower in tumor samples than in normal samples (**Figure 3A**). Meanwhile, survival analysis showed that LUAD patients with high GPR65 expression had longer OS than those with low LUAD expression (**Figure 3B**; *p* = 0.003), while both GPR174 and MS4A1 were highly expressed in tumor samples (**Figures 3C, E**), and LUAD patients with high expression of GPR174 and MS4A1 had a better survival prognosis than the low expression group (**Figures 3D, F**; *p* = 0.002, *p* < 0.001). In addition, TIMD4 expression was not significantly different between tumor samples and normal tissues (**Figure 3G**) and not correlated significantly with OS of patients (**Figure 3H**; *p* = 0.142). The above results suggest that there may be a positive effect of GPR65 to inhibit LUAD progression. Therefore, we chose to focus our analysis on GPR65.

### 3.6 Association between GPR65 expression and clinicopathological characteristics and disease progression

**Figure 4:**
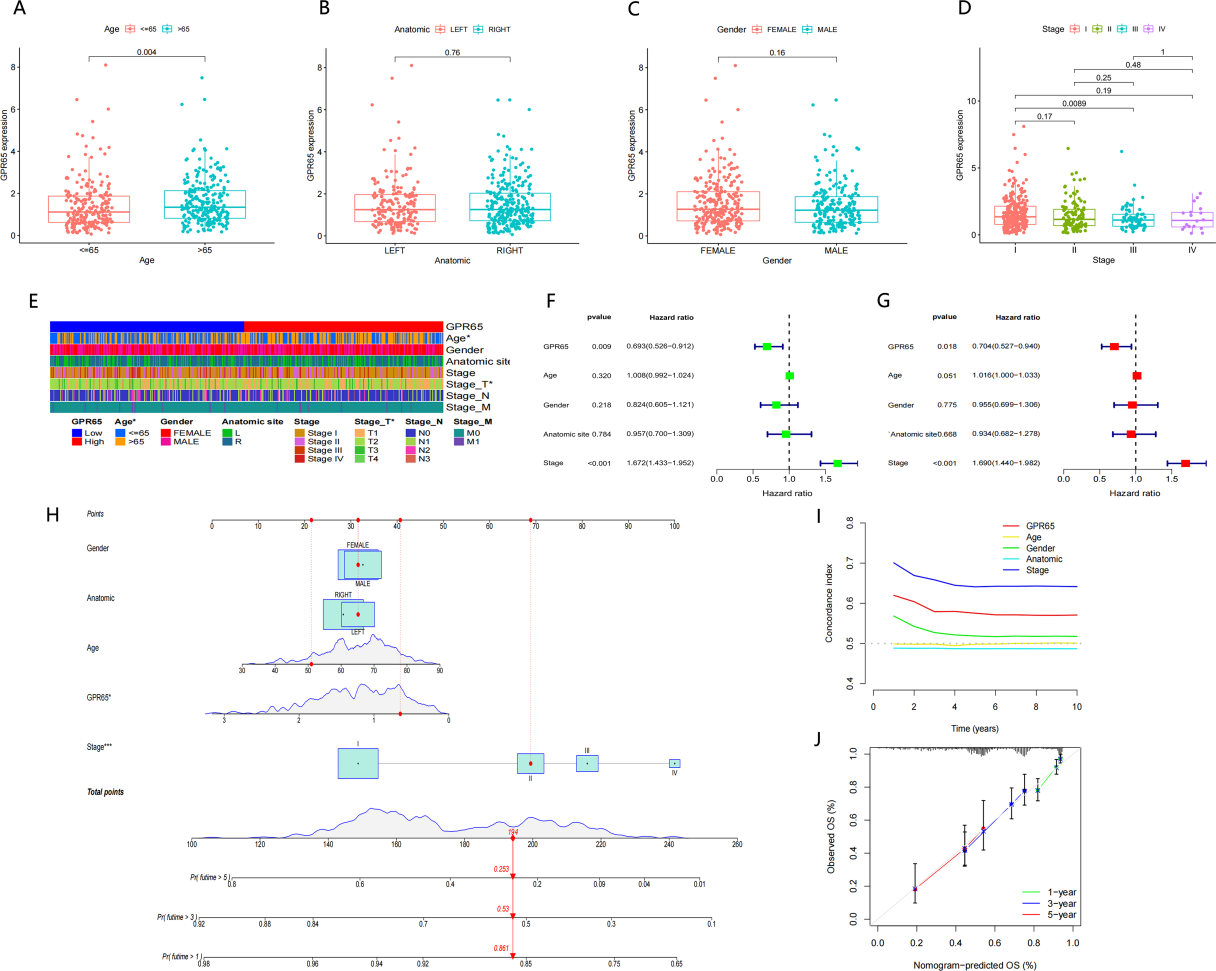
Association between GPR65 expression and clinicopathological characteristics. Boxplots show that **(A)** Age is significantly associated with GPR65 expression, but other plots have no statistical difference with GPR65 **(B–E)** Strip chart showed that Age and Stage_T were significantly associated with GPR65. **(F-G)** Univariate and multivariate Cox regression analyses of GPR65 expression and four other clinicopathological parameters in the TCGA-LUAD cohort. **(H)** Nomogram of GPR65 and four other clinicopathological characteristics in the diagnosis of LUAD patients. **(I)** Time dependent C-index curves of the GPR65 and four other clinical traits. **(J)** Calibration graphs indicated that predicted 1,3, and 5 year survival rates were close to the actual survival rates.

We set out to explore the relationship between the GPR65 gene and clinicopathological features. The results showed that GPR65 expression differed significantly between different age groups (**Figure 4A**; *p* = 0.004). However, there was no significant difference in the expression level of GPR65 between anatomic (**Figure 4B**; *p* = 0.76), gender (**Figure 4C**; *p* = 0.16) and stage (**Figure 4D**; *p* = 0.19). As shown in **Figure 4E**, GPR65 was significantly associated with age and T stage. To further explore the prognostic predictive ability of GPR65 expression, we performed univariate cox regression analysis (**Figure 4F**; Hazard Ratio [HR]: 0.693, 95% Confidence Interval [95%CI]: 0.526-0.912; *p* = 0.009) and multivariate cox regression analysis (**Figure 4G**; HR:0.704, 95%CI: 0.527-0.940; *p* = 0.018) with complete information on age, gender, anatomic site, and stage. The results showed that both GPR65 and stage were powerful and independent prognostic predictors of OS. Based on the GPR65 and the above 4 clinical information profiles, we created a nomogram model to predict the prognosis of patients (**Figure 4H**). The C-index of the five indicators in nomogram was shown in **Figure 4I**, and the calibration curve showed that the prediction results of the nomogram model were basically the same as the ideal model (**Figure 4J**), which could provide an idea for clinicians to predict the survival rate of patients at 1, 3 and 5 years. These results indicated that the clinical prognostic model constructed based on GPR65 could effectively predict the survival prognosis of LUAD patients.

### 3.7 GO and KEGG profiles indicate that GPR65 is enriched in immune-related pathways

To further explore the mechanism of the role of GPR65 gene in LUAD progression, we performed GO enrichment analysis of GPR65, which showed significant enrichment in antigen binding (MF), T cell receptor complex (CC) and leukocyte-mediated immunity (BP) (**Supplementary Figure 3A**). Meanwhile, GPR65 was also closely related to immune response pathways such as cytokine-cytokine receptor interaction and chemokine signaling pathway (**Supplementary Figure 3B**). Next, to further validate this observation, we used GSEA to find enriched pathways in the KEGG dataset, and **Supplementary Figure 3C** showed visually the top 5 Hallmarks, where the GPR65 high expression group is mainly enriched in immune-related signaling pathways, such as allograft rejection, interferon gamma response, while the GPR65 low expression group was mainly enriched in MYC_targets_V2, pancreas_beta_cells, and unfolded_protein_response pathways. In addition, **Supplementary Figure 3D** displayed visually the top 5 pathways, Parkinsons_disease, Ribosome, Spliceosome, Maturity_onset_diabetes_of_the_young were differentially enriched in GPR65 low expression samples, and only Olfactory_ transduction was differentially enriched in GPR65 high expression samples. These results provide evidence for phenotypes that may be involved in GPR65 expression.

### 3.8 GPR65 enhances infiltration of immune activated cells and enhances immunotherapeutic effects

**Figure 5:**
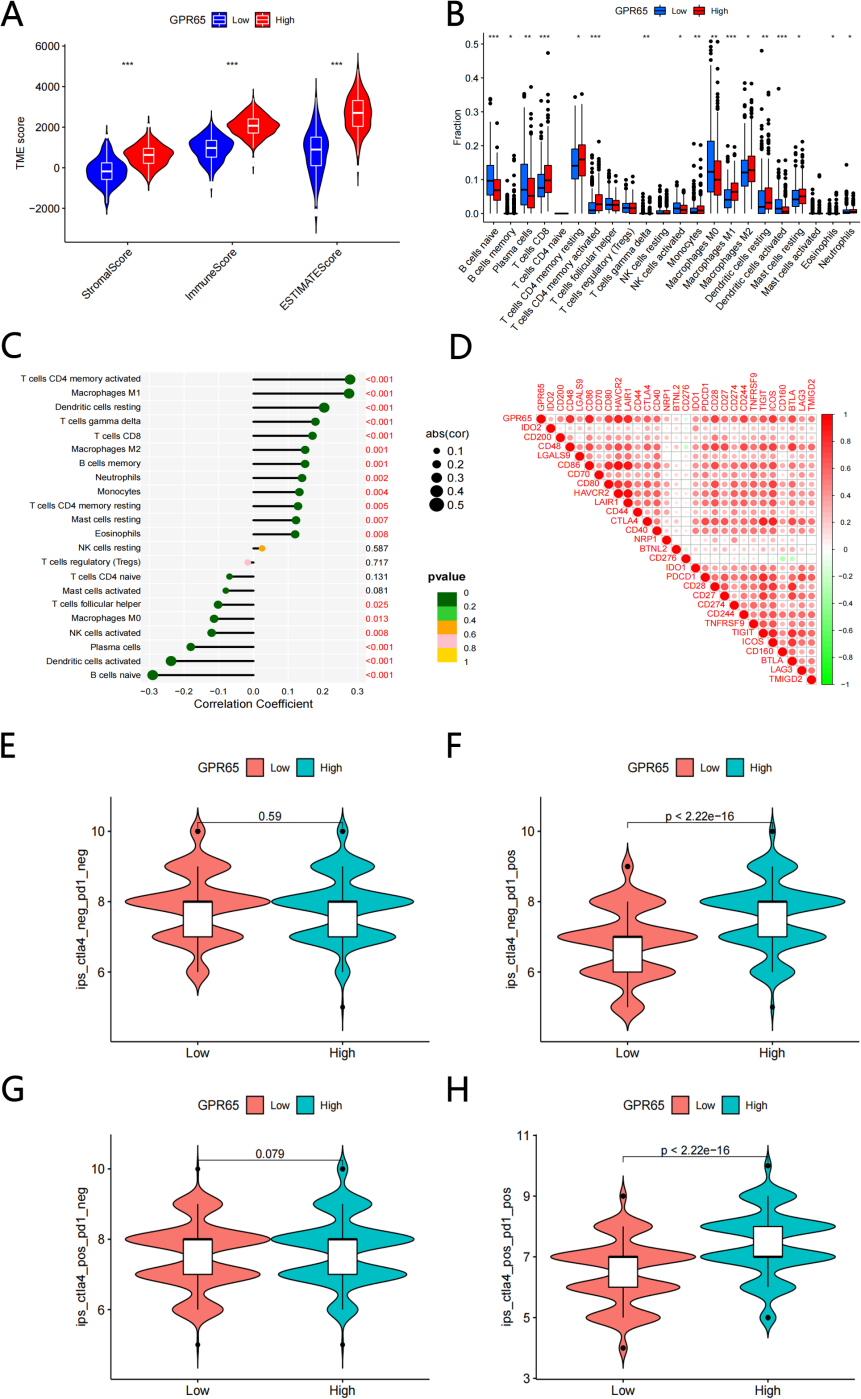
GPR65 were associated with immune cell infltration and immune checkpoint. **(A)** Comparisons of the levels of three kinds of TME scores analysis in the high and low GPR65 groups**(B)** Diferential fractions of 22 immune cells in low and high-expressed groups. **(C)** Relationship between 22 immune cell types by CIBERSORT. **(D)** The correlation between 29 immune checkpoint-related genes and GPR65. **(E-H)** GPR65 high expressed were positively correlated with upregulated programmed cell death protein 1 (PD1) level and cytotoxic T lymphocyte-associated antigen-4 (CTLA4) plus PD1 level, whereas the other plots showed no statistical difference in patients with LUAD.

The GPR65 high and low expression groups were closely correlated with the ImmuneScore, StromalScore and ESTIMATEScore, and in all three score systems, the high GPR65 expression group had a higher TME Score than the low expression group (**Figure 5A**). Next, we analyzed the difference in the level of immune cell infiltration between the high and low GPR65 expression groups. The infiltration levels of Memory B cells, CD8+ T cells, Memory resting CD4+ T cells, Memory actived CD4+ T cells, Resting Dendritic cells, Resting Mast cells, Eosinophils, and Neutrophils in the high-risk group were significantly higher than those in the low-risk group, on the contrary, Naive B cells, Plasma cells, T cells gamma delta, Active NK cells, Macrophages M0, Active Dendritic cells were significantly elevated in the low-risk group (**Figure 5B**). To further explore the relationship between GPR65 and immune cell infiltration, we used correlation coefficient to describe, the coefficients and significance of GPR65 in 22 immune-related subpopulations are shown in **Figure 5C**. We found that GPR65 gene was closely associated with TIIC subpopulations, which indicated that GPR65 gene was mainly involved in the immune response during LUAD progression. To determine the relationship between GPR65 and immune checkpoint molecules, we analyzed the correlation between GPR65 and immune checkpoint molecules expression (**Figure 5D**). ICI therapy, represented by CTLA-4/PD-1 inhibitors, has certainly made a major breakthrough in antitumor therapy. In addition to the well-known tumor mutation burden (TMB), PD-L1 and MSI, the newly identified predictor IPS is strongly recommended for the assessment of immune response. Our analysis also showed that PD1_IPS and PD1+CTLA4 score was significantly higher in the GPR65 high expression group (**Figures 5F, H**; both *p* < 0.05). However, there was no significant correlation between the high and low GPR65 risk subgroups in the CTLA4 and no PD1+CTLA4 studies (**Figures 5E, G**; both *p* > 0.05). In summary, these results could indicate that GPR65 could benefit patients in anti-PD1 immunotherapy for LUAD patients.

### 3.9 GPR65 is clinically and cellularly validated as suppressor gene

**Figure 6:**
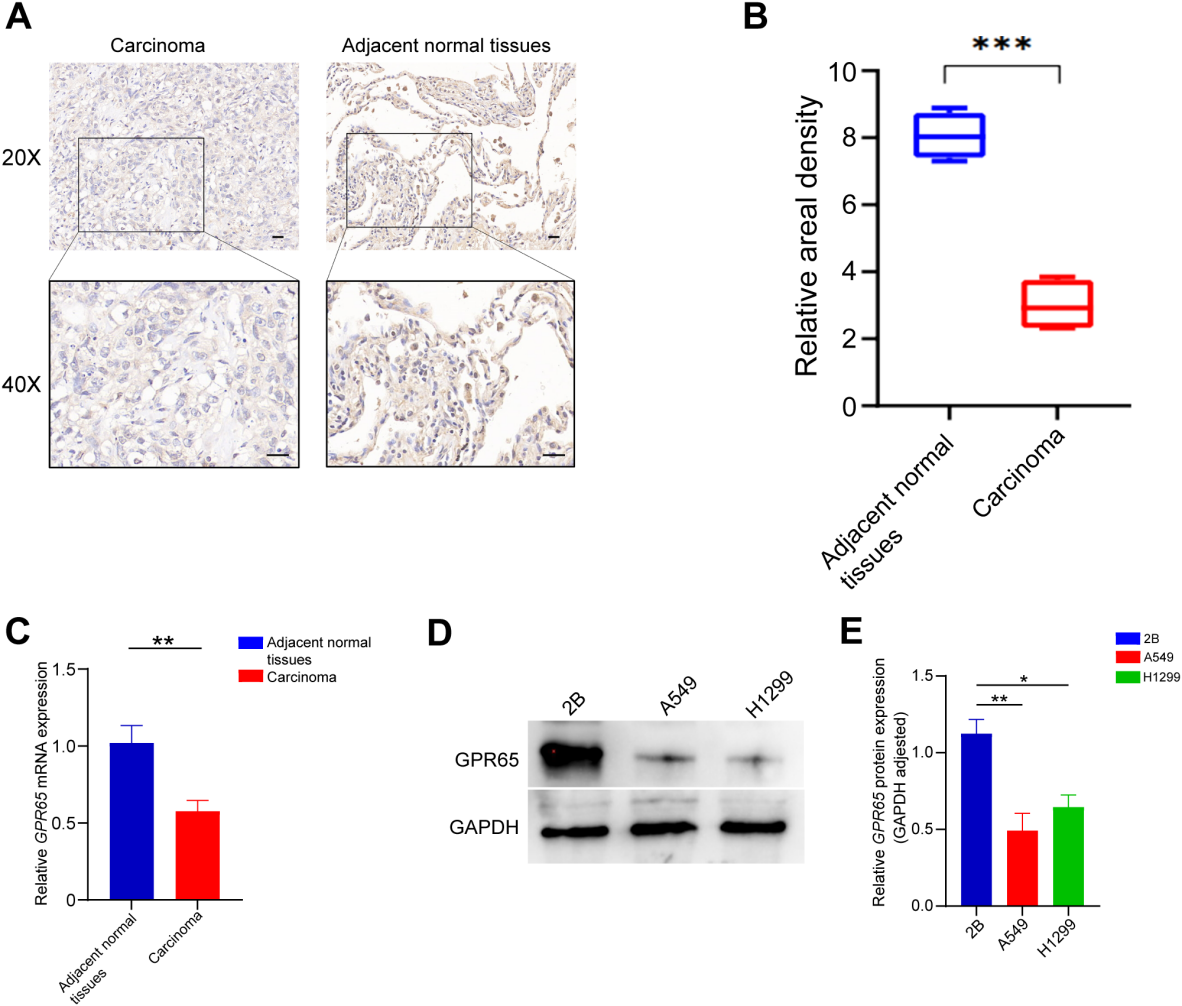
GPR65 is low expressed in both LUAD tissues and cell lines. **(A-B)** GPR65 was low expressed in LUAD tissues compared to adjacent normal tissues; **(C)** Low mRNA expression of GPR65 in LUAD tissues compared to adjacent normal tissues; **(D-E)** Low expression of GPR65 in LUAD cells compared to normal alveolar epithelial cells.

To reveal the role of GPR65 in LUAD, immunohistochemistry staining was performed on LUAD tissues and adjacent normal tissues. **Figure 6A** depicts a higher GPR65 expression in the adjacent normal tissues, and a lower level of expression in the LUAD tissues. IHC staining scores showed significantly lower expression of GPR65 in LUAD compared to adjacent normal tissues (**Figure 6B**). QRT-PCR data showed that GPR65 mRNA expression levels were significantly reduced in LUAD tissues compared to normal tissues (**Figure 6C**). The difference in GPR65 expression between normal lung epithelial cell lines and two LUAD cell lines were verified by Western blot analysis (**Figures 6D, E**). Although the H1299 cell line was not statistically significant, it showed the same expression trend as the A549 cell line. All the above experimental results indicated that the expression level of GPR65 was decreased in LUAD patients, which could be used as a potential indicator of poor clinical prognosis.

### 3.10 Up-regulation of GPR65 attenuates neoplasm proliferation and invasiveness *in vitro*

**Figure 7:**
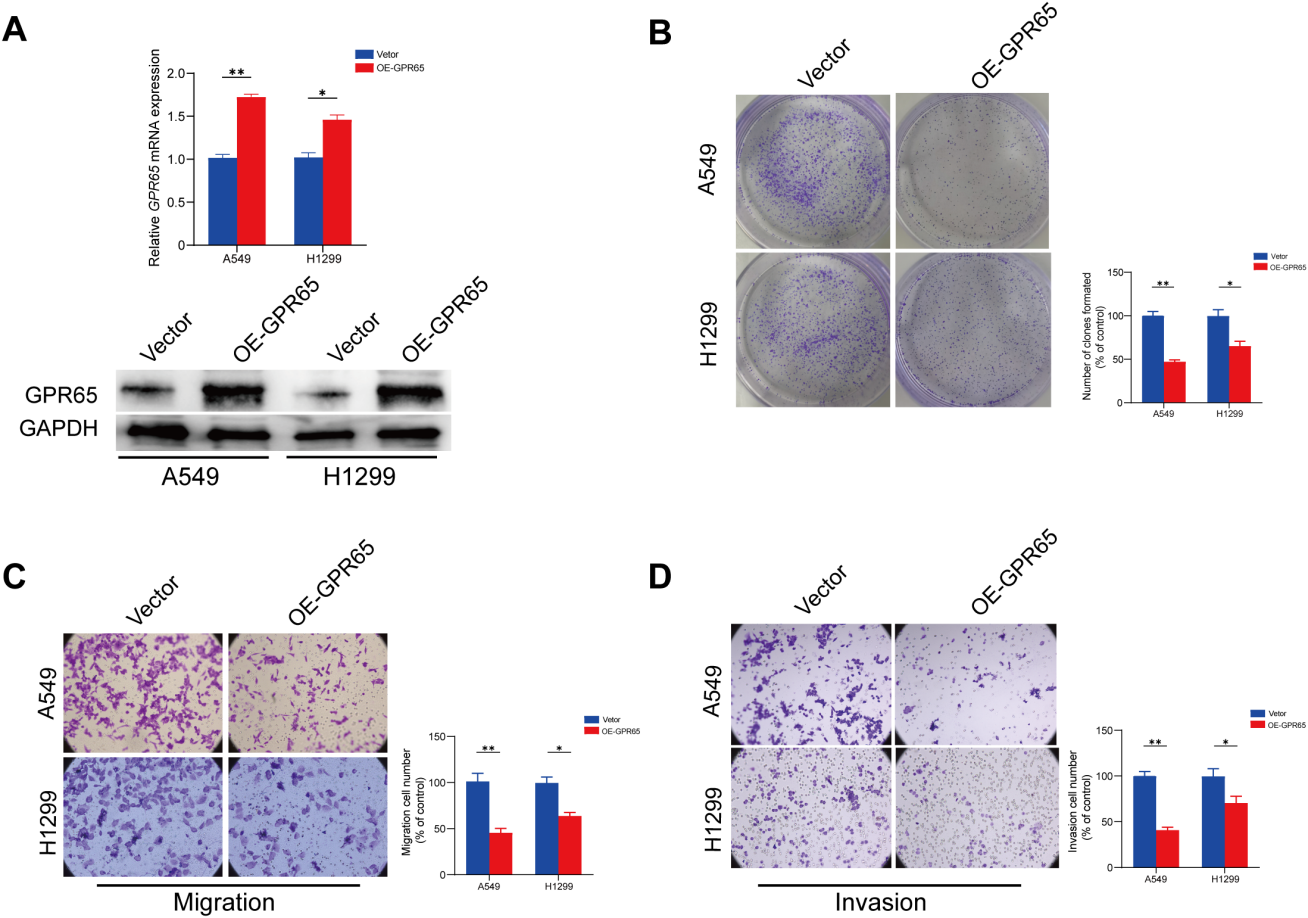
Up-regulation of GPR65 attenuates proliferation and invasiveness in vitro. **(A)** Clone formation assays performed with A549 and H1299 cells; **(B-C)** Representative images of indicated cells migration and invasion treated with medium from vetor lung cancer cells or si-GPR65. **(D)** Representative images of indicated cells invasion treated with medium from vetor lung cancer cells or OE-GPR65.

To authenticate the influence of GPR65 on proliferation and invasion *in vitro*, we selected two cell lines, A549 and H1299, with moderate GPR65 content to examine the effect of GPR65 on proliferation and invasion. In this study, clone formation, transwell assay, was used to assess the ability of tumor cells to proliferate and invade. We found that knockdown of GPR65 in lung cancer cells significantly increased the proliferation of A549 and H1299 compared to the control (Vector) (**Figure 7A**). Moreover, in the transwell assay, knockdown of GPR65 enhanced the migration and invasion of A549 and H1299 cells, respectively (**Figures 7B–C**). On the other hand, overexpression of GPR65 enhanced these abilities of tumor cells (**Figure 7D**). These results support the notion that GPR65 may be involved in the regulation of lung cancer cell proliferation and invasion.

### 3.11 GPR65 inhibits lung adenocarcinoma cell progression via the JAK2/STAT3 axis

**Figure 8:**
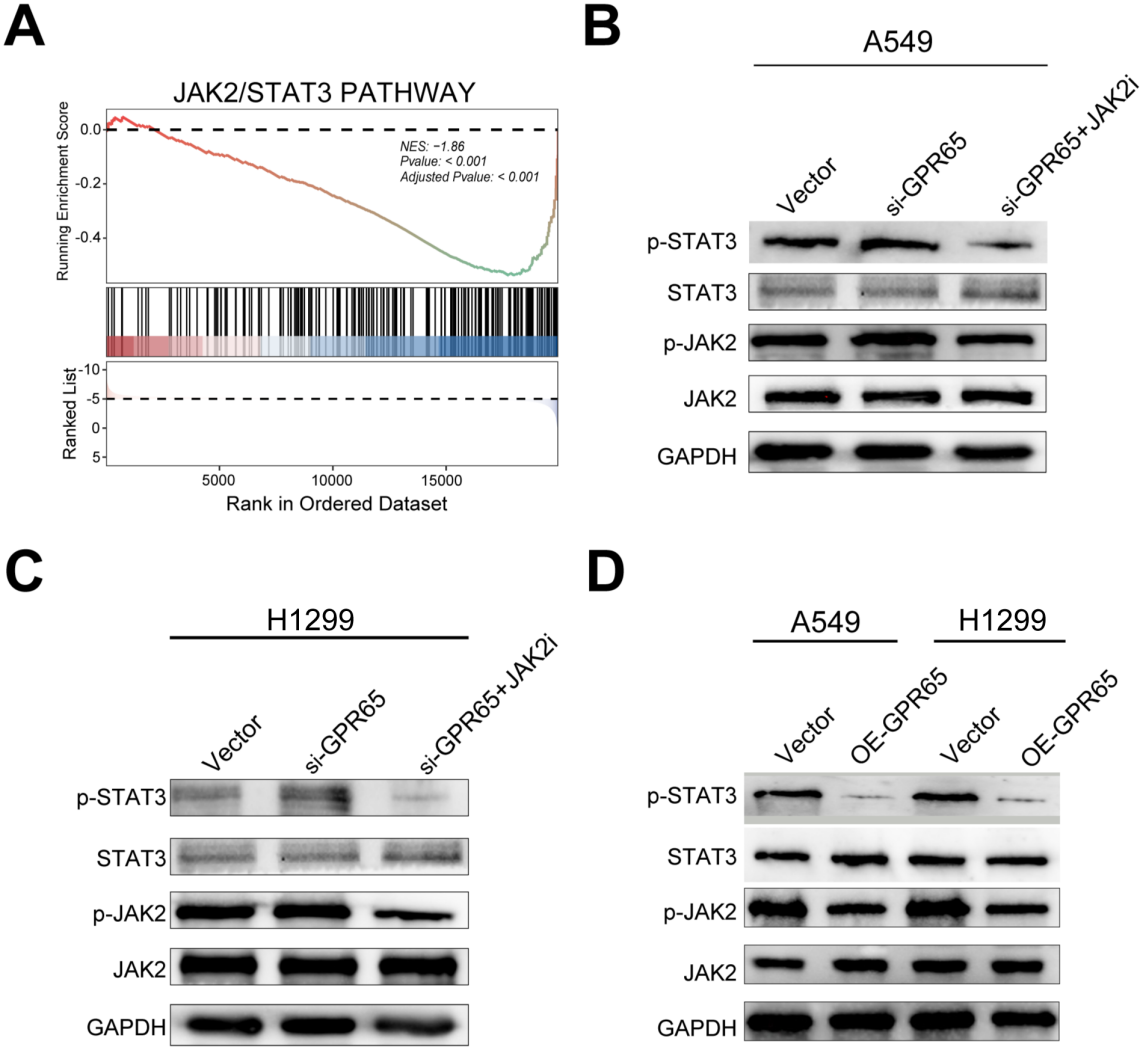
GPR65 inhibits tumors through the JAK2/STAT3 signaling pathway. **(A)** GSEA analysis of GPR65 in the TCGA database. **(B-C)** Immunoblot analysis of p-JAK2 and JAK2 as well as p-STAT3 and STAT3 expression in shCtr cells and siGPR65 cells with or without JAK2 inhibitor. gAPDH was used as an internal control. **(D)** Immunoblotting was performed to analyze the expression of p-JAK2 and JAK2 as well as p-STAT3 and STAT3 in overexpressing GPR65 cells. GAPDH was used as an internal control.

The malignant progression of LUAD is regulated by multiple signaling pathways, such as STAT3, ERK, AKT and EGFR pathways. Previous KEGG analysis suggested that GPR65 regulates the JAK2/STAT3 pathway, therefore, we performed GSEA analysis of GPR65 by using TCGA database, which showed that GPR65 negatively regulates the JAK2/STAT3 signaling pathway (**Figure 8A**). To examine whether JKA2 and STAT3 are regulated by GPR65, the results showed that knockdown of GPR65 in LUAD cells upregulated p-JAK2 and p-STAT3 expression without affecting the total levels of JAKA2 and STAT3; however, it was rescued by the addition of a JAK2 inhibitor (**Figures 8B–C**). Furthermore, overexpression of GPR65 in LUAD cell lines decreased the p-JAK2 and p-STAT3 expression rather than increasing the total levels of JAK2 and STAT3 (**Figure 8D**).

### 3.12 GPR65 overexpression inhibits tumorigenesis *in vivo*

**Figure 9:**
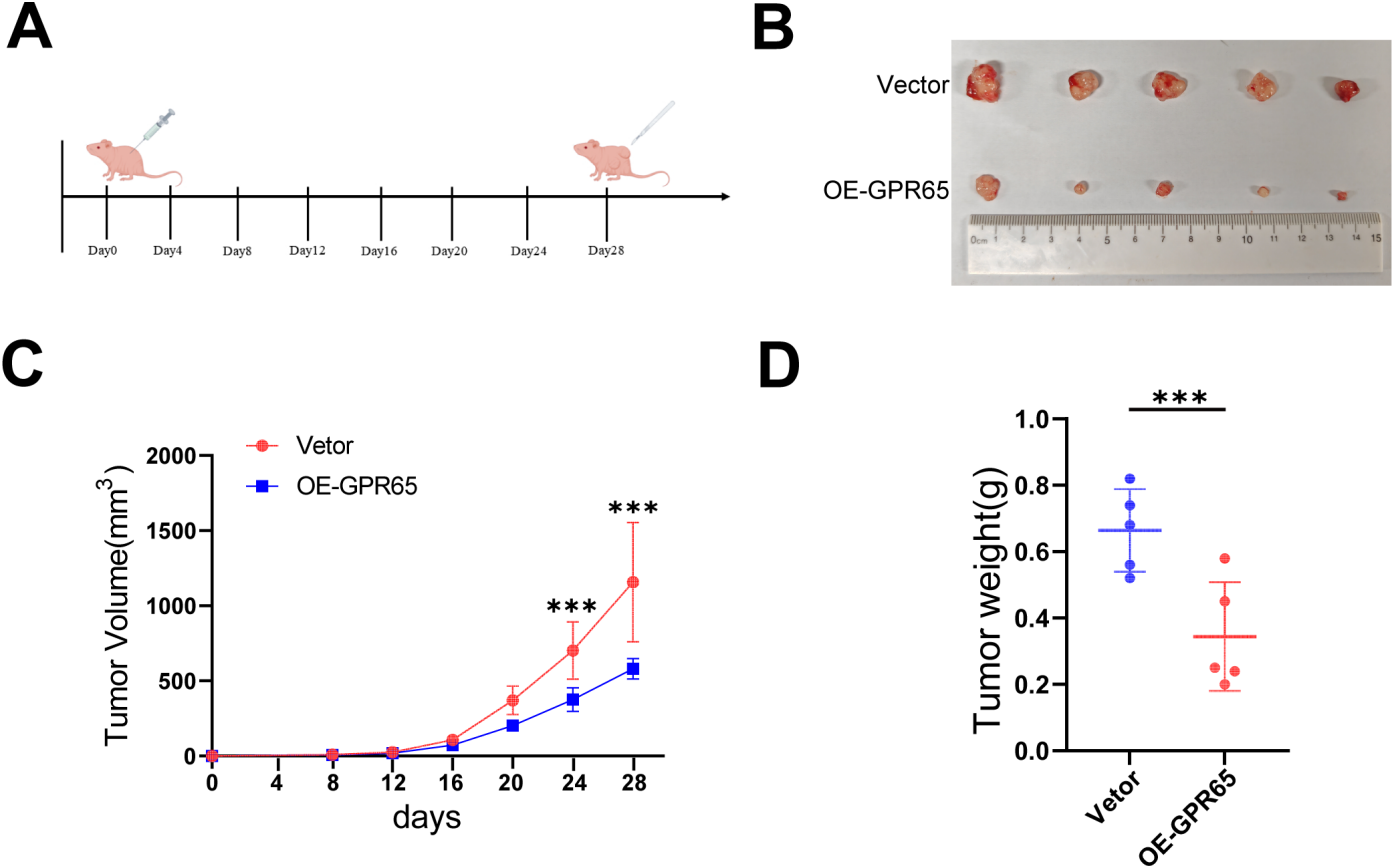
Overexpression of GPR65 inhibits tumorigenesis *in vivo*. **(A)** Schematic diagram of subcutaneous tumor formation in nude mice; **(B)** Representative tumors are shown; **(C) **Tumor growth curves depicting tumor volume over time; **(D)** Scatter plot of individual tumor weights at the experimental endpoint.

To investigate the role of GPR65 in tumorigenesis *in vivo*, we inoculated A549-shGPR65 cells into the flanks of nude mice (**Figure 9A**). At the end of the experiment, these animals were executed and the tumors were dissected and weighed (**Figure 9B**). Tumors from GPR65 overexpressing tumor cells were smaller and lighter (**Figures 9C–D**). Collectively, these data suggest that GPR65 is an oncogene *in vivo* that inhibits tumor growth.

Translated with www.DeepL.com/Translator (free version)

## 4 Discussion

GPR65, also known as T-cell death-associated gene 8 (TDAG8), is a glycosphingolipid receptor and a member of the module co-varying with pro-inflammatory genes (24, 25). GPR65 is expressed in many cancer types, including colon cancer, hematologic malignancies, melanoma, etc. As an acid-sensitive proton-sensing G protein-coupled receptor (GPCR), GPR65 plays an important role in the development and progression of chronic inflammatory diseases (19), autoimmune diseases (26), and even tumors in human.

TME has long been considered a typically acidic environment due to inadequate blood perfusion, hypoxia, inflammation, and glycolytic cellular metabolism, and the unique glycolytic metabolism of cancer cells produces excess lactic acid, which can lead to acidosis in TME (18, 27–30). GPR65, a member of the pH-sensing GPCR family, can help cells sense extracellular acidosis (31). It has been found that the acidic microenvironment may induce PD-L1 on cancer cells and thus down-regulate anti-tumor immune responses, while in human Head and Neck squamous cell carcinoma (HNSCC), PD-L1 mRNA expression correlated with GPR65 mRNA expression. In addition, extracellular acidic pH could activate GPR65 to transactivate multiple downstream G protein signaling pathways, such as: Gα13 G protein/Rho GTPase signaling pathway (20, 32). Interestingly, the Gα13 G protein/Rho GTPase signaling pathway showed completely opposite differential effects in different cancer types: pro-tumorigenic in various types of epithelial cancers but anti-tumorigenic in hematological malignancies. In another study, GPR65 and its downstream PKA pathway were engaged in the perception of cancer pain in rats (33).

In this study, we found that GPR65 was lowly expressed in LUAD and significantly positively correlated with the prognosis of LUAD. By correlation analysis between GPR65 and clinicopathological features, we found that the expression of GPR65 gene was higher in the early stage of LUAD than in the middle and late stages, which suggested that the heterogeneity within the tumor might suppress the expression of this gene in the late stage of LUAD. In addition, we found that GPR65 could be an independent prognostic factor for LUAD, and based on this, we drew a nomogram and accurately predicted the 1-, 3-, and 5-year OS for LUAD. It was reported that GPR65 may be involved in regulating tumor progression through TME (18). Therefore, we further analyzed the relationship between GPR65 expression and TME. KEGG results showed that the high GPR65 expression group was mainly enriched in immune-related signaling pathways, such as allograft rejection and interferon response; while the low GPR65 expression group was mainly enriched in MYC_ targets_V2, pancreatic beta cells and unfolded protein response. Unfolded protein response (UPR), a highly regulated mechanism of signal transduction, protects cells from accumulation of misfolded proteins, not only modifying inappropriate proteins but also degrading unrecoverable proteins, the dysregulated UPR may play a crucial role in cancer (34–36). MYC family transcription factors play a key role in the development and progression of human cancers, and alterations in MYC oncogenes are a hallmark of many human cancers, supporting the process and progression of tumor, and elevated MYC levels are also associated with resistance to therapy (37–39). Currently, it has been shown that there is a complex interaction between MYC and UPR signaling, and activation of both may jointly promote tumor progression (40). These evidences further suggest that GPR65 has important research value as a potential biomarker.

It has been shown that GPR65 can enhance tumor progression by promoting the adaptation of cancer cells to an acidic environment and by facilitating cell survival and proliferation. An earlier study found that GPR65 was overexpressed in colon, ovarian and kidney tumor tissues (22). Another study showed that overexpression of GPR65 in Lewis lung cancer cells stimulated tumor cell growth and may promote resistance to acidosis-mediated cell death *in vitro* via protein kinase A (PKA) and ERK-related pathways (41). However, our results showed that GPR65 expression was instead reduced in LUAD patients, which seemed to be inconsistent with the expression results in most cancer types. Whereas, a study conducted by Calvin R Justus et al. showed that GPR65 gene expression was detrimental to the growth of acute myeloid leukemia cells compared to normal blood cells and tissues. GPR65 gene expression is usually downregulated in hematologic malignancies (42). This is somewhat consistent with our findings of low GPR65 gene expression in LUAD patients. In addition, they performed additional bioinformatics analysis of the tumor database and found an interesting phenomenon: GPR65 expression was either unaltered or downregulated in lung cancer samples compared to normal lung tissue. This phenomenon also supported our findings. Further corroboration came from a series of studies that GPR65 has multiple pro- and oncogenic effects (43–45), These effects are cancer type and environment dependent, and these seemingly contradictory observations can be further explained by the Gα13 G protein/Rho GTPase signaling described above. Thus, GPR65 may play a two-sided role in tumors, either promoting survival or inducing apoptosis. Given its multiple functions, it may prove to be a promising target for immunotherapy.

In the present study, analysis of the relationship between GPR65 expression and immune cell types in LUAD samples showed that Memory B cells, CD8+ T cells, Memory resting CD4+ T cells, Memory activated CD4+ T cells, Macrophages M1 and Macrophages M2, etc. were significantly and positively correlated with GPR65 expression. This is consistent with our hypothesis that GPR65 may be a key gene associated with survival prognosis in LUAD. Previous studies showed that B cells can promote differentiation of tumor-specific CD4+ T follicular helper cells in a neoantigen-dependent manner, which in turn enhanced CD8+ T cell effector function through IL-21 production and drove anti-tumor immunity in a mouse lung adenocarcinoma model (46). A recent study also revealed a correlation between the number of tumor-infiltrating B cells and the survival of LUAD patients with specific mutation drivers, suggesting that tumor-infiltrating B cells may be a marker for cell-specific mutations in lung cancer (47). In addition, several studies have suggested that T cells and macrophages are closely associated with the clinical results of LUAD (48). In conclusion, immune cell infiltration may play a subtle but crucial role in LUAD progression. Therefore, we should delve into its specific molecular regulatory mechanisms to better evaluate the prognosis of patients.

In addition, we tried to determine the correlation between GPR65 expression levels and the expression levels of immune checkpoint molecules. The results showed that GPR65 expression was positively correlated with most immune checkpoints, suggesting that it may be used in some ways to improve the efficacy of immunotherapy. This was further validated in the TCIA analysis, where the GPR65 high expression cohort could benefit from treatment with PD1, but failed to benefit from treatment with CTLA4. However, further experimental validation of the exact process was still needed.

Of course, the present study has some limitations that must be acknowledged. First, since the clinical data were mainly obtained from the TCGA database, bias in the results is inevitable and we still need to further validate these findings through *in vivo* experiments. Moreover, we did not clarify the specific ways and mechanisms by which key genes affect tumor immunity and exert anti-tumor effects. Lastly, a significant limitation is the context-dependent duality of GPR65, functioning as potentially tumor-suppressive in some contexts (like our findings in LUAD and reports in hematologic malignancies) while appearing oncogenic in others (e.g., colon, ovarian, kidney cancers).

## 5 Conclusions

>Our research comprehensively and systematically analyzed the involvement of GPR65 in the expression, diagnostic value, survival prognostic value, immune infiltration, and possible mechanism of LUAD. In conclusion, our results provide new markers and therapeutic targets for LUAD patients and may provide useful information for accurately predicting the prognosis and treatment of LUAD patients.

## Data Availability

The original contributions presented in the study are included in the article/**Supplementary Material**. Further inquiries can be directed to the corresponding authors.

## Supplementary material

Supplementary Figure 1 Correlation of clinicopathological characteristics with immune, stromal, and estimate scores. **(A-D)** Distribution of ImmuneScore in Age, Anatomic site, Gender and Stage (Wilcoxon rank sum test). **(E-H)** Distribution of StromalScores in Age, Anatomic site, Gender and Stage (Wilcoxon rank sum test). **(I-L)** Distribution of ESTIMATEScores in Age, Anatomic site, Gender and Stage (Wilcoxon rank sum test).

Supplementary Figure 2 Construction of risk signature in the TCGA cohort. **(A)** Cross-validation for tuning the parameter in the LASSO regression. **(B)** LASSO coefficient profiles of the 16 survival-related genes.

Supplementary Figure 3 Functional enrichment analyses of GPR65. **(A)** Bubble graph for GO enrichment (larger numbers in the bubbles mean more enriched genes). **(B)** The enriched KEGG pathways based on GPR65 (increasingly significant enrichment from blue to red). **(C-D)** Top 5 enriched Hallmarks and KEGG pathways associated with GPR65.
