# Supplementary figures and images for: GPR65 is a novel immune biomarker and regulates the immune microenvironment in lung adenocarcinoma
Source: Front Immunol. 2025 May 30;16:1572757. doi: 10.3389/fimmu.2025.1572757 (PMC12162609; doi:10.3389/fimmu.2025.1572757)

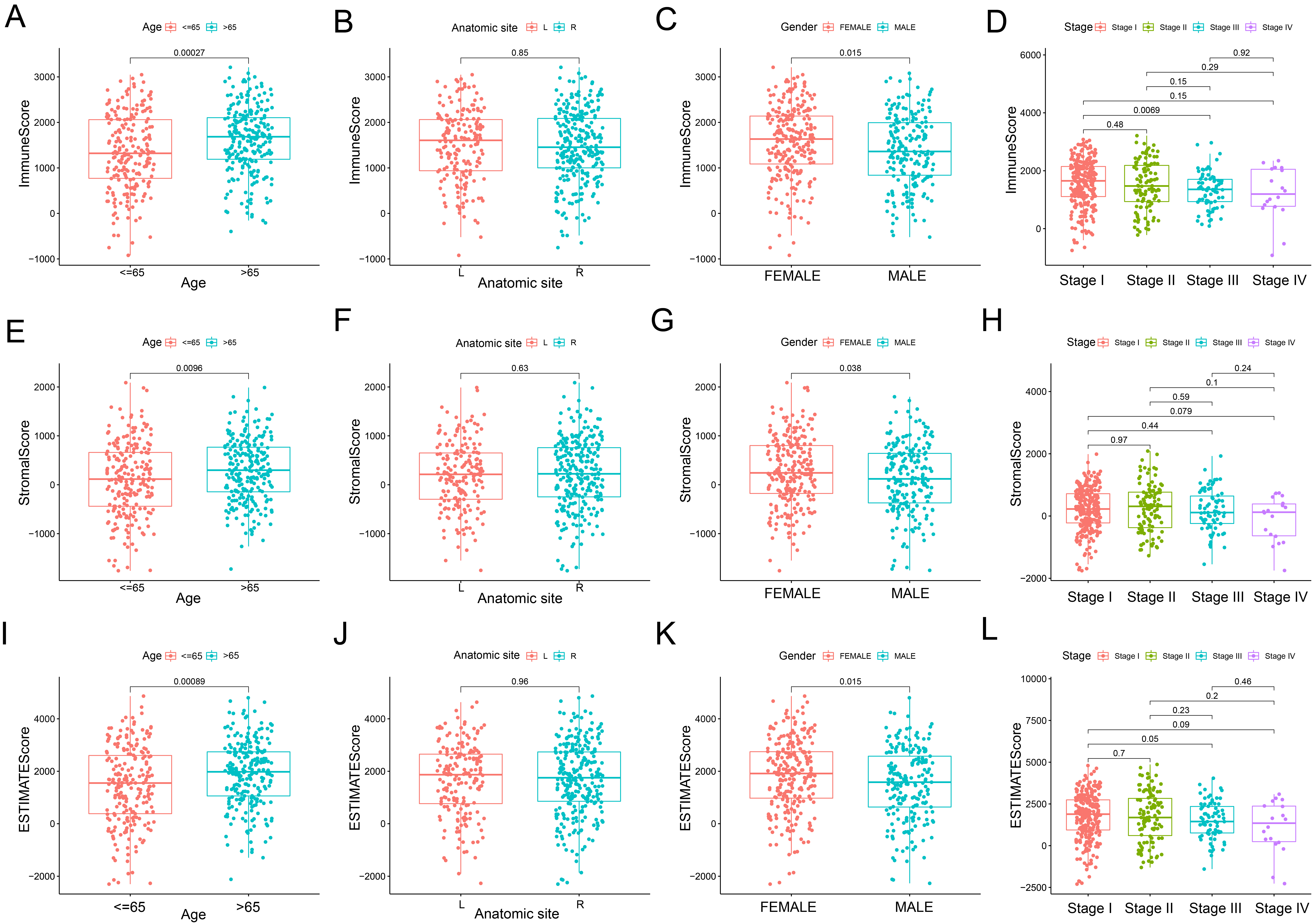

Supplement: Supplementary Figure 1 — Correlation of clinicopathological characteristics with immune, stromal, and estimate scores. (A-D) Distribution of ImmuneScore in Age, Anatomic site, Gender and Stage (Wilcoxon rank sum test). (E-H) Distribution of StromalScores in Age, Anatomic site, Gender and Stage (Wilcoxon rank sum test). (I-L) Distribution of ESTIMATEScores in Age, Anatomic site, Gender and Stage (Wilcoxon rank sum test). [file Image1.tiff]

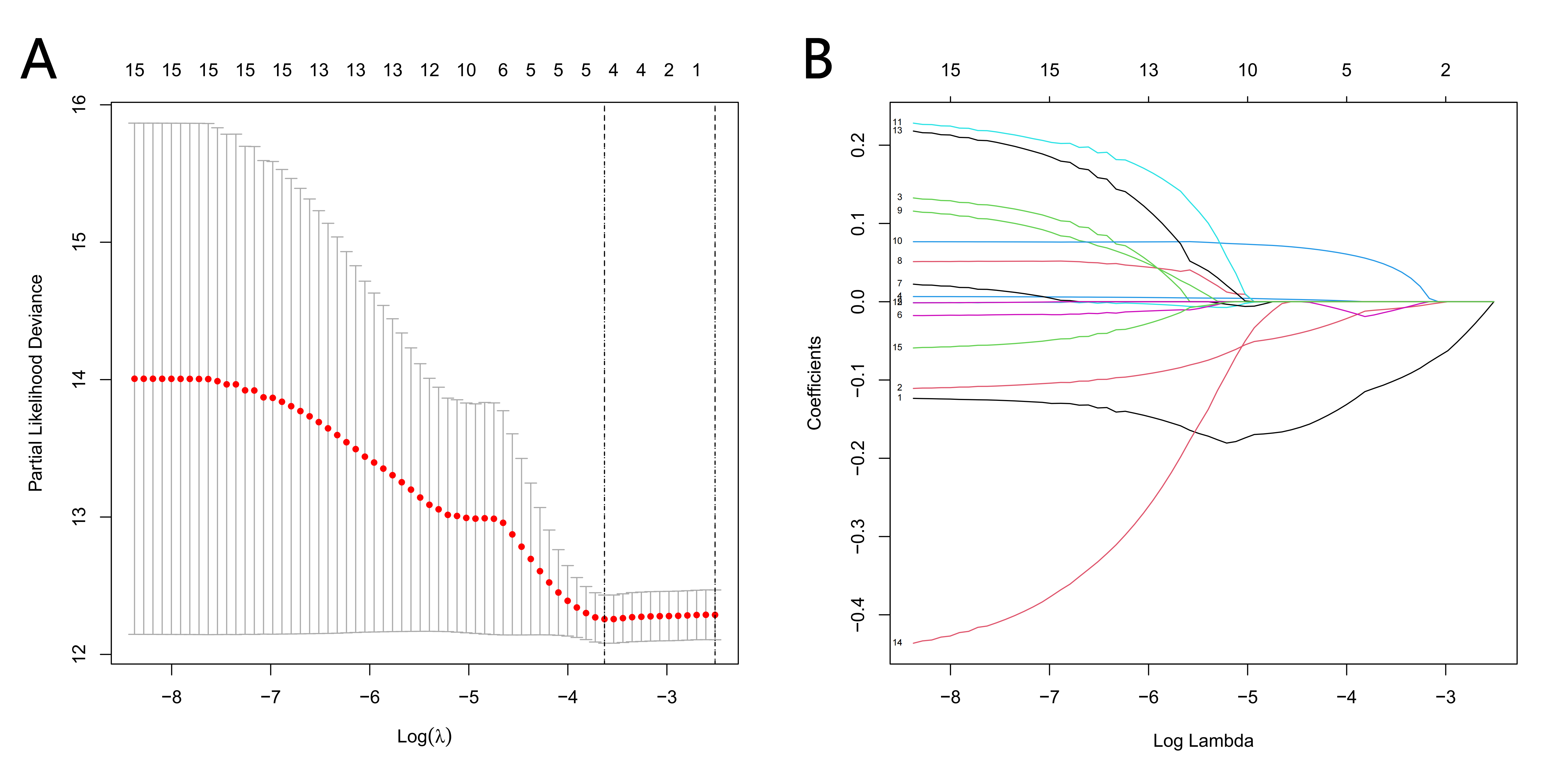

Supplement: Supplementary Figure 2 — Construction of risk signature in the TCGA cohort. (A) Cross-validation for tuning the parameter in the LASSO regression. (B) LASSO coefficient profiles of the 16 survival-related genes. [file Image2.tiff]

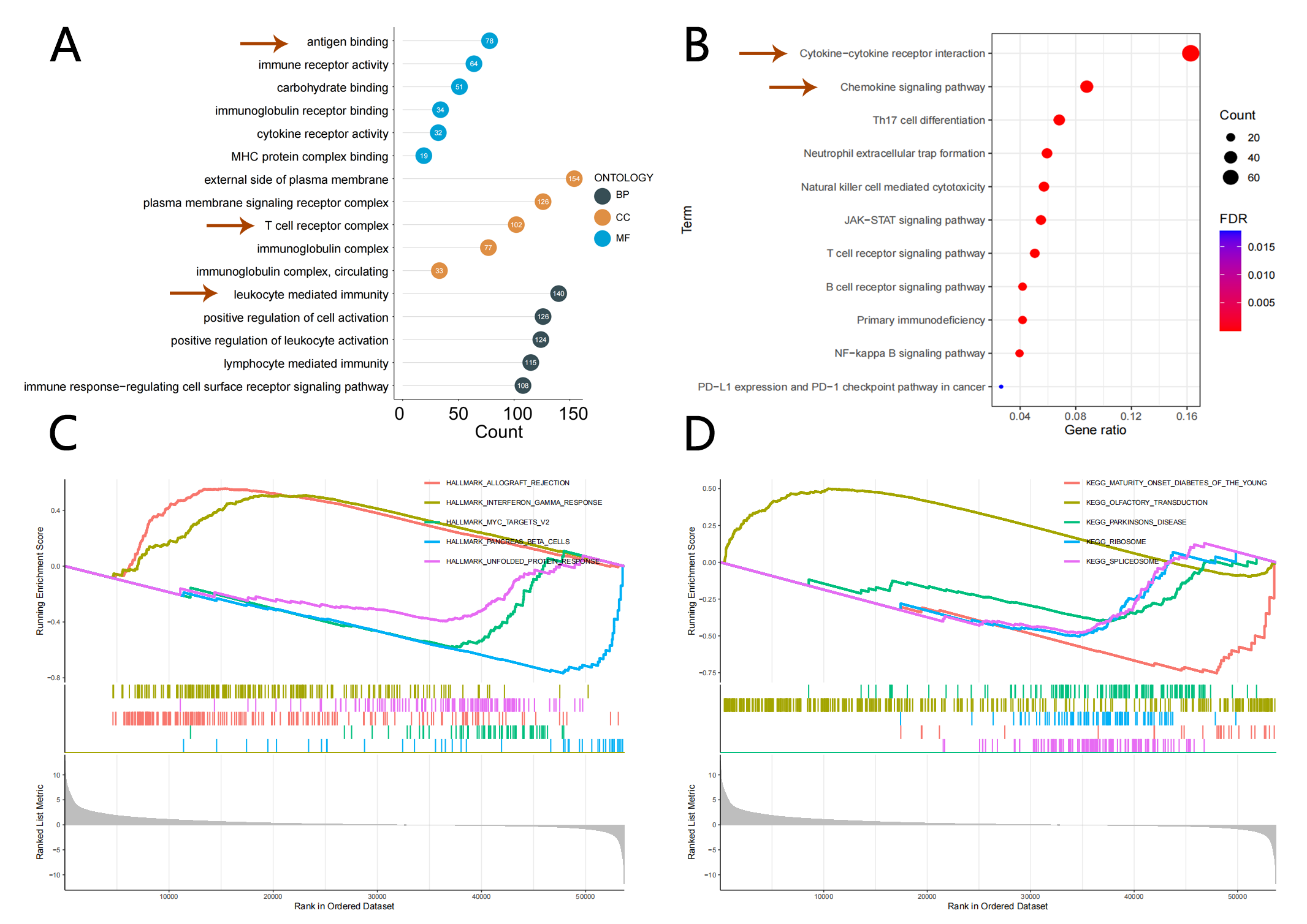

Supplement: Supplementary Figure 3 — Functional enrichment analyses of GPR65. (A) Bubble graph for GO enrichment (larger numbers in the bubbles mean more enriched genes). (B) The enriched KEGG pathways based on GPR65 (increasingly significant enrichment from blue to red). (C-D) Top 5 enriched Hallmarks and KEGG pathways associated with GPR65. [file Image3.tif]

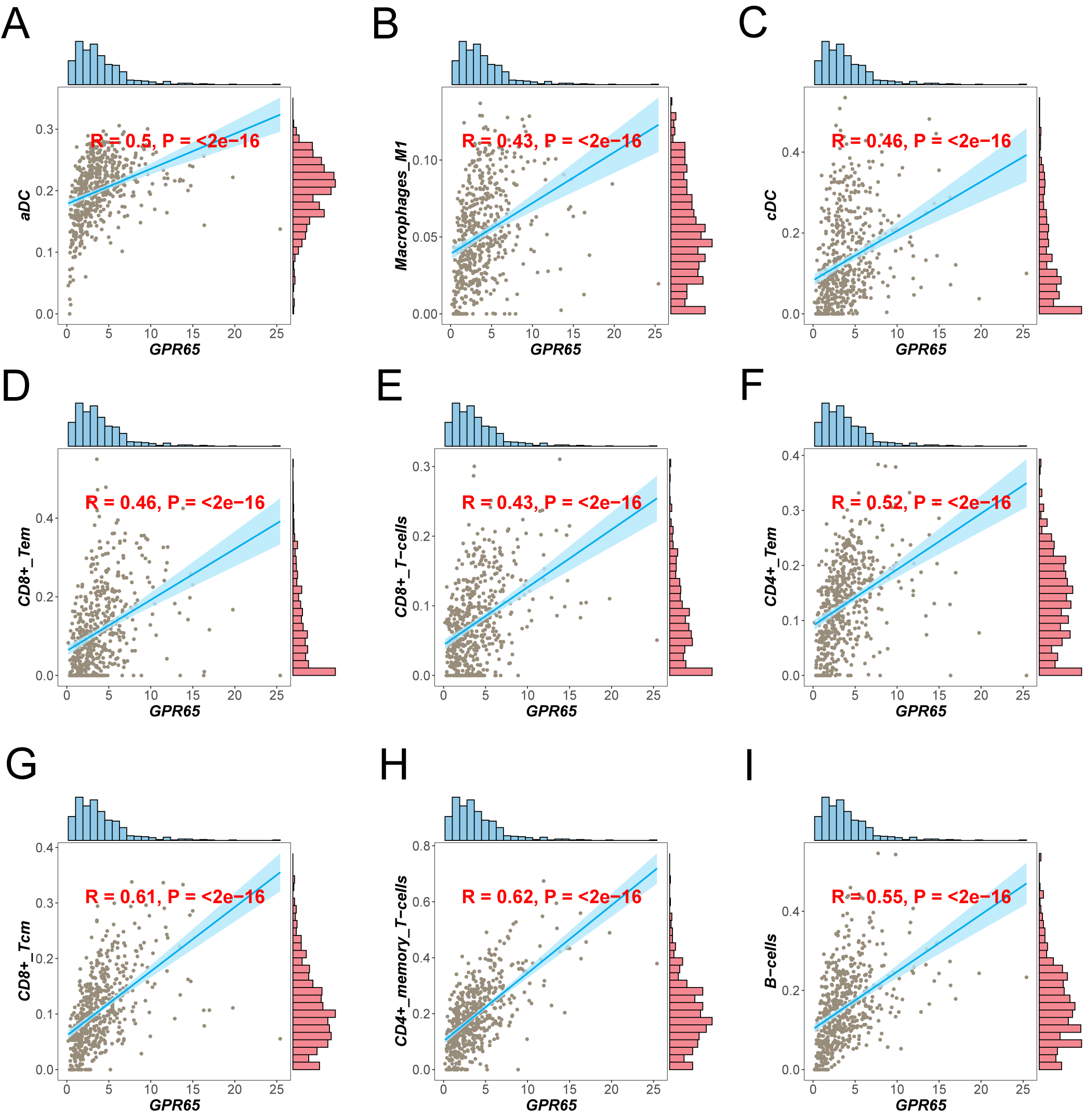

Supplement: Supplementary file 4 [file Image4.tif]

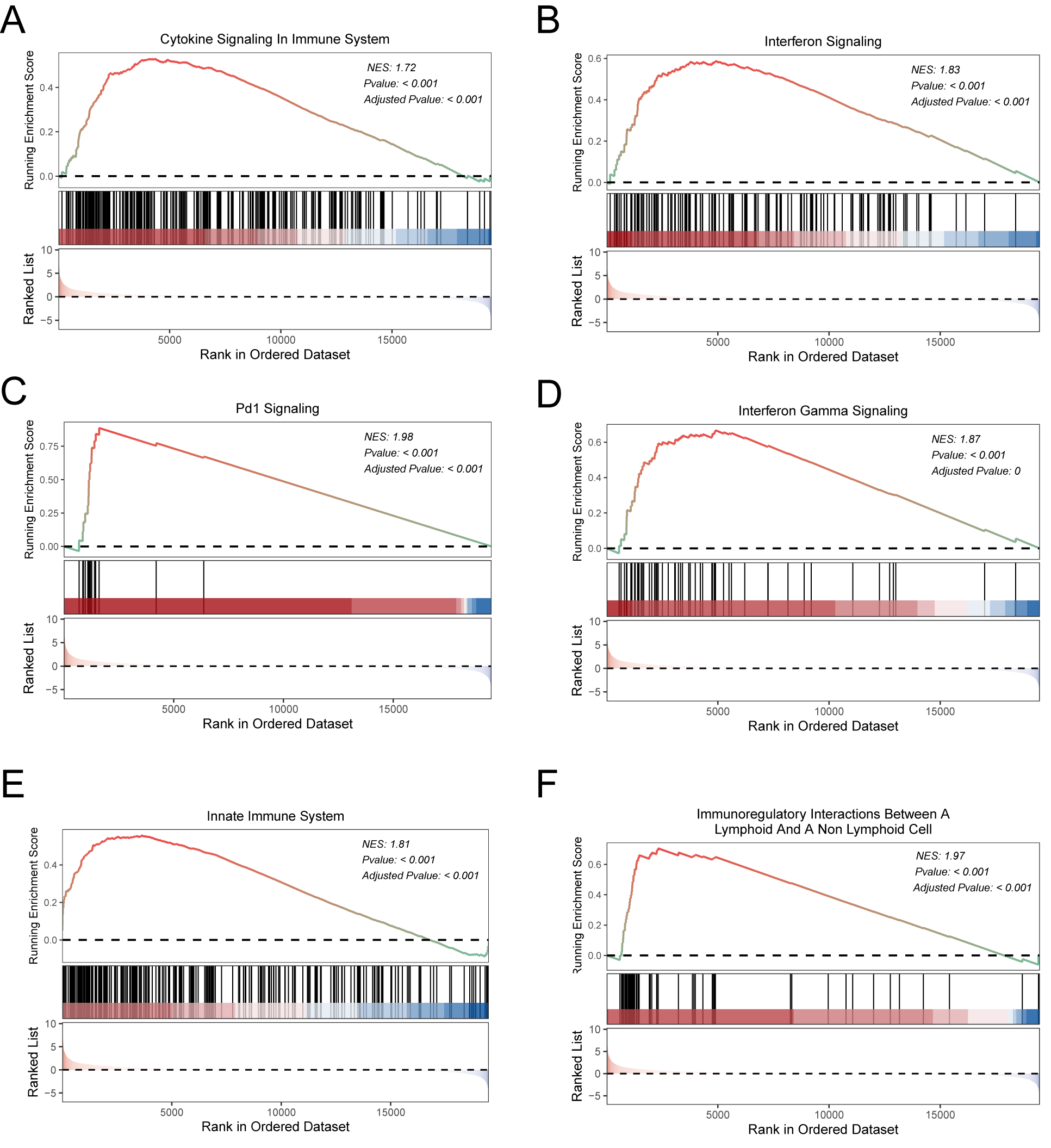

Supplement: Supplementary file 5 [file Image5.tif]

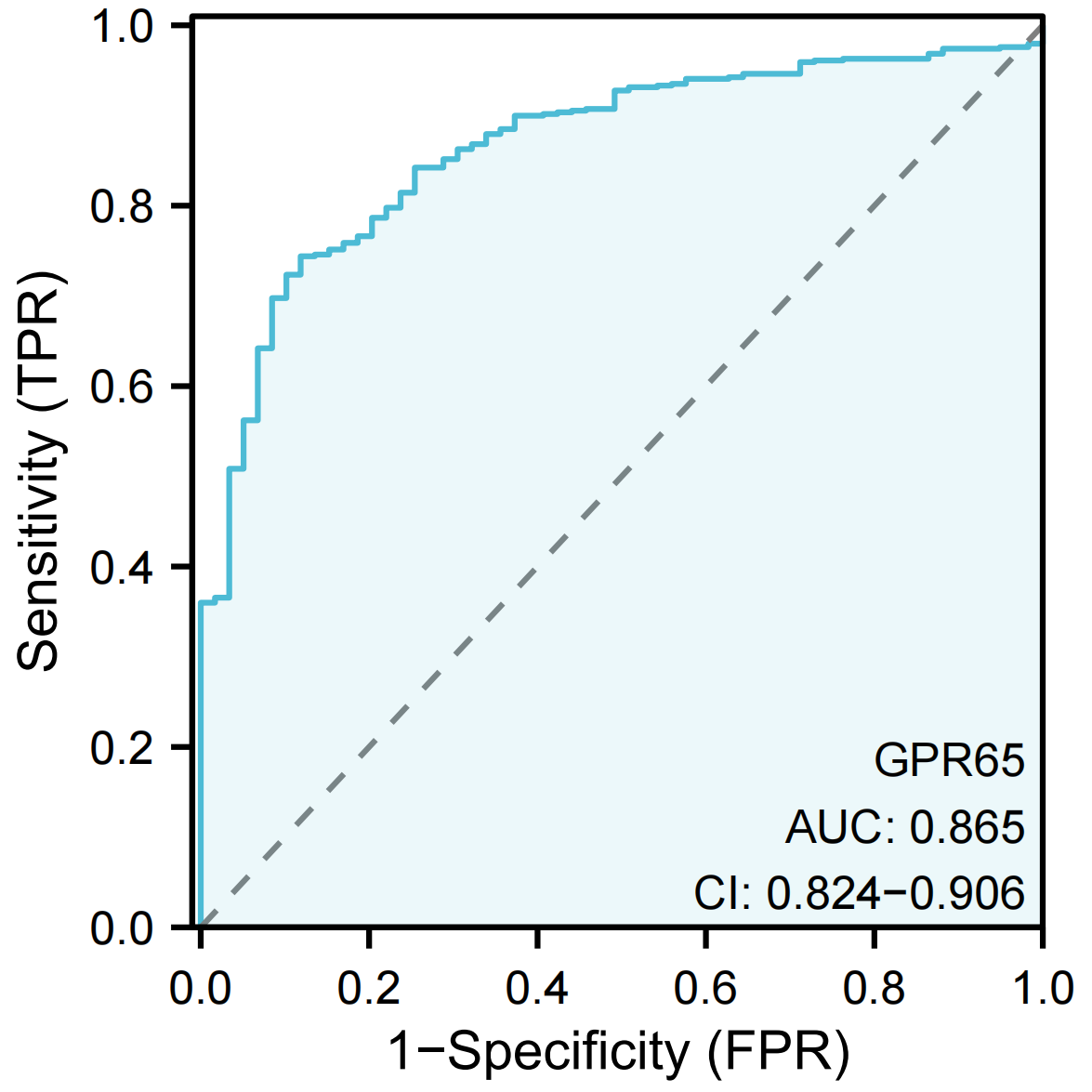

Supplement: Supplementary file 6 [file Image6.tif]

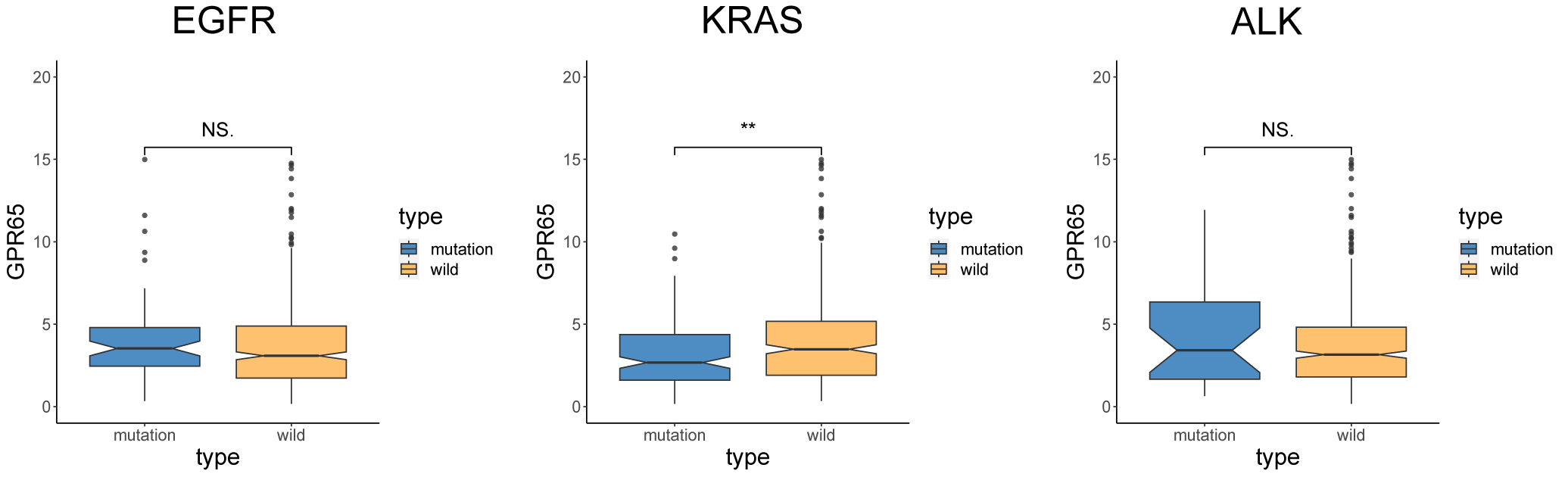

Supplement: Supplementary file 7 [file Image7.tif]

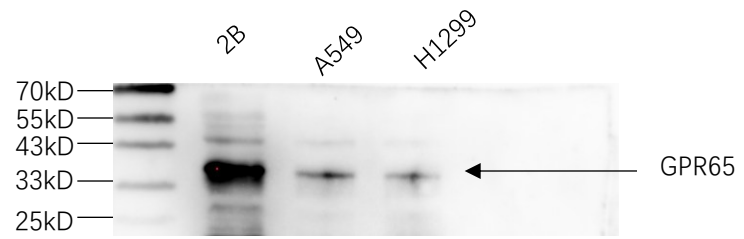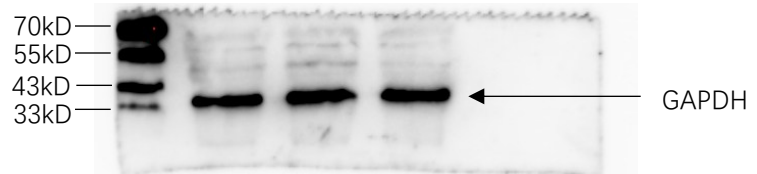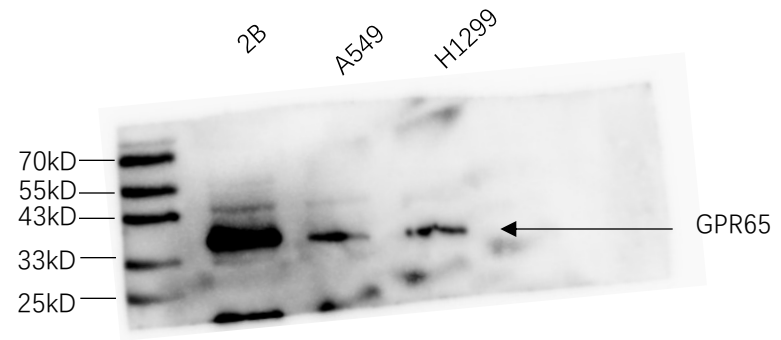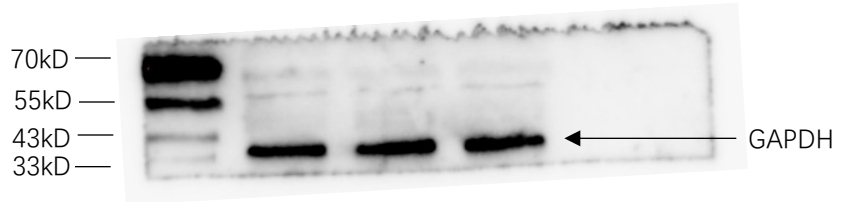

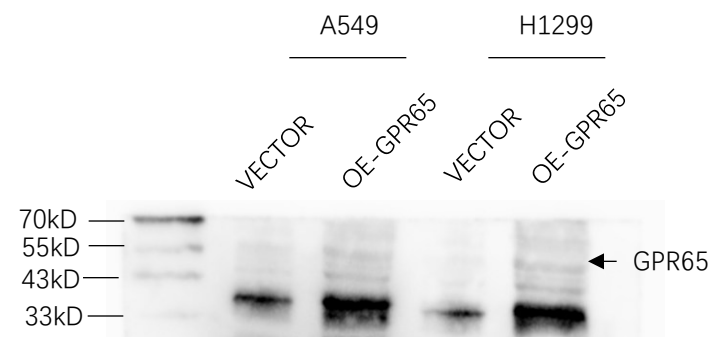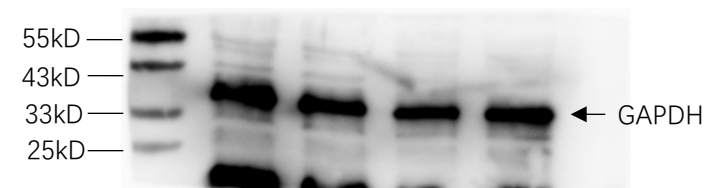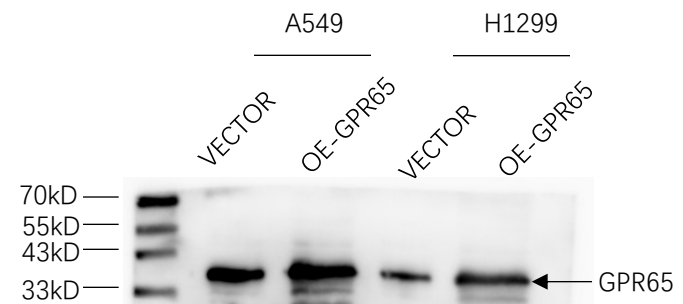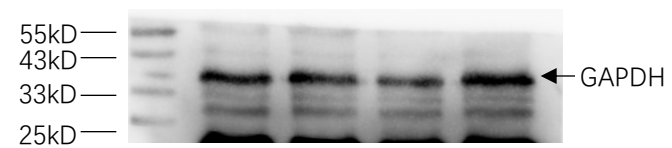

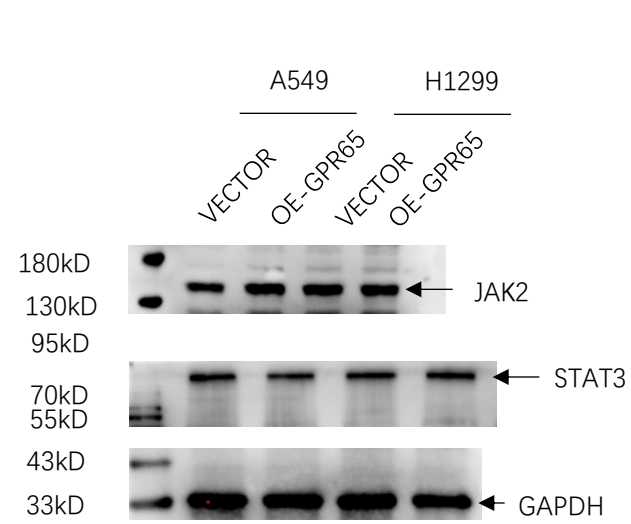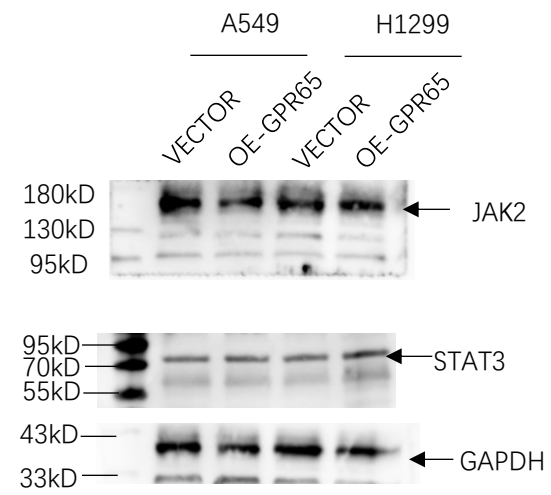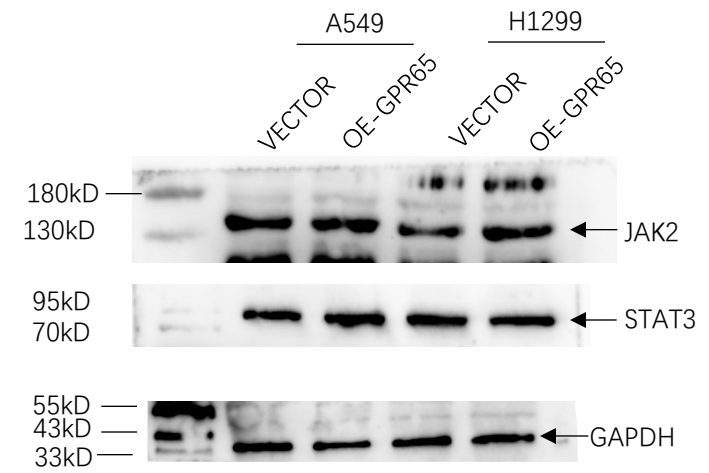

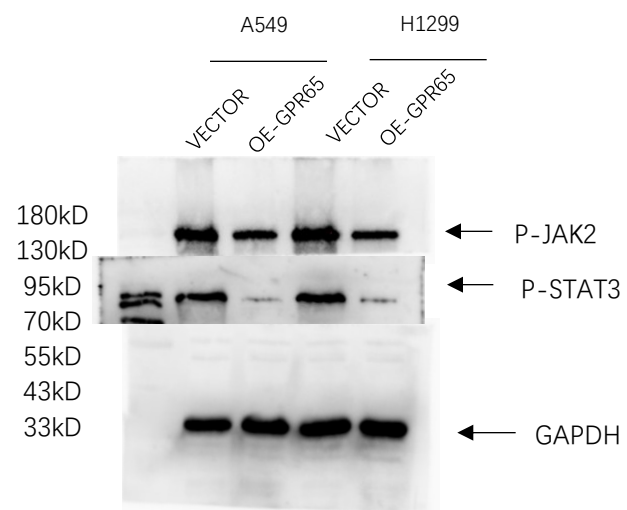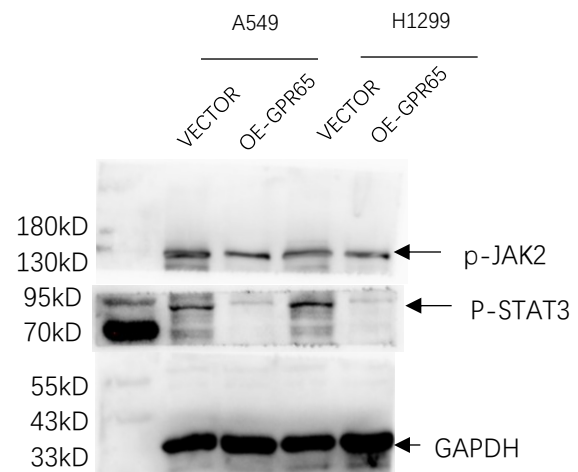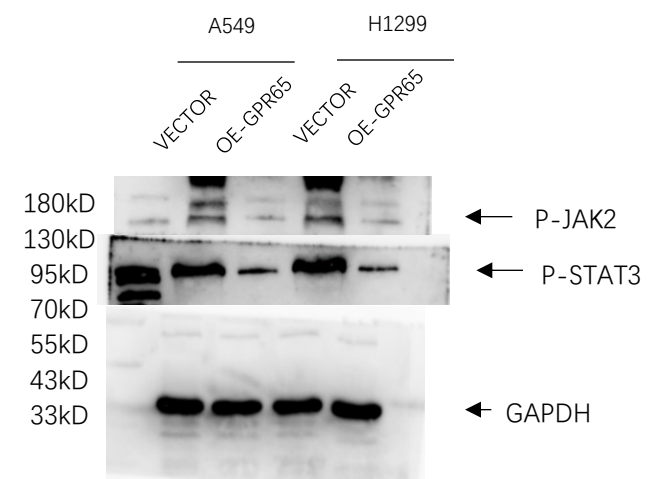

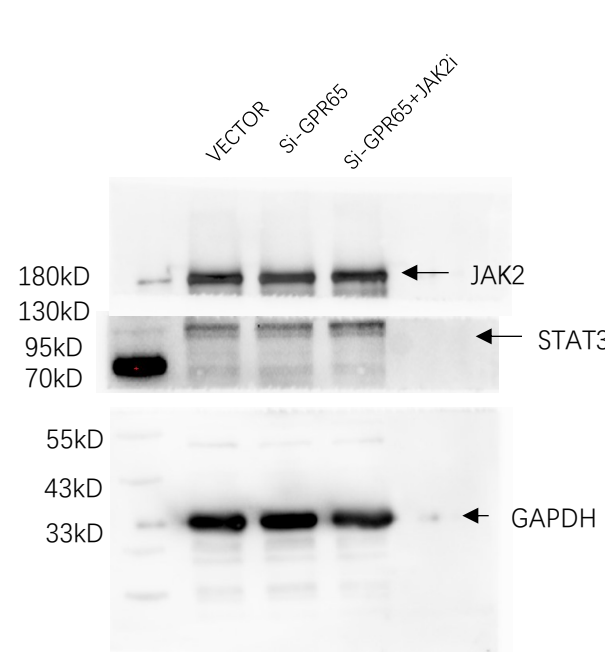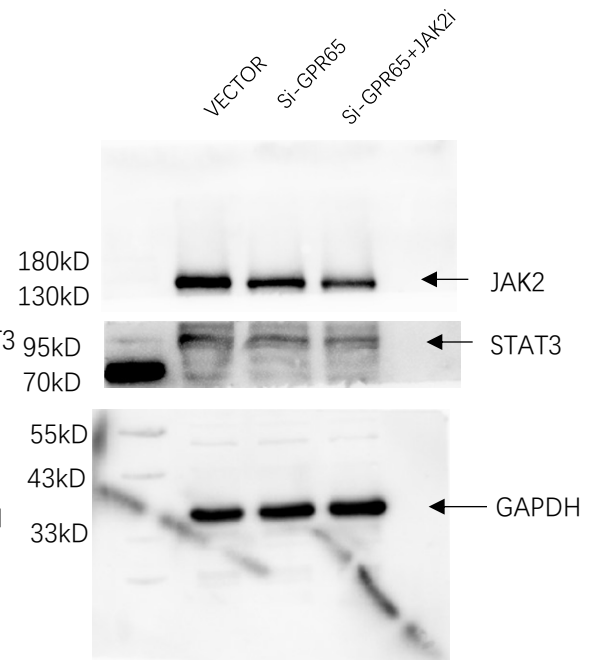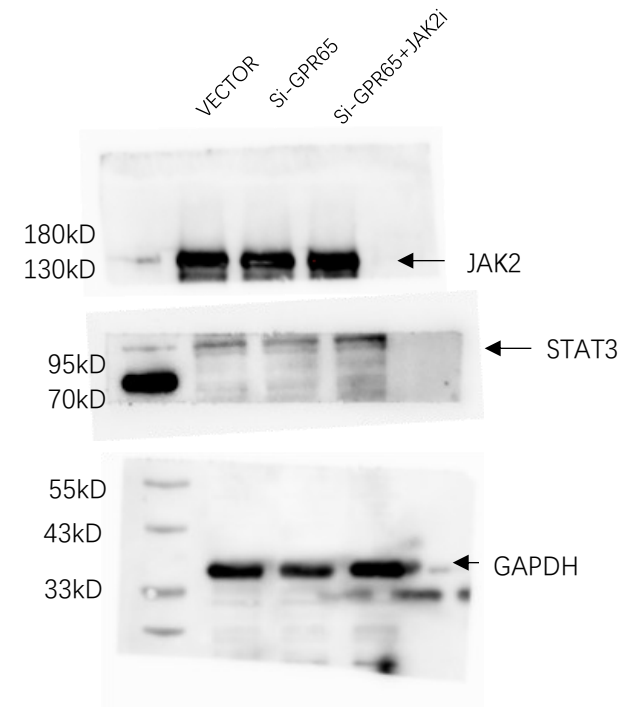

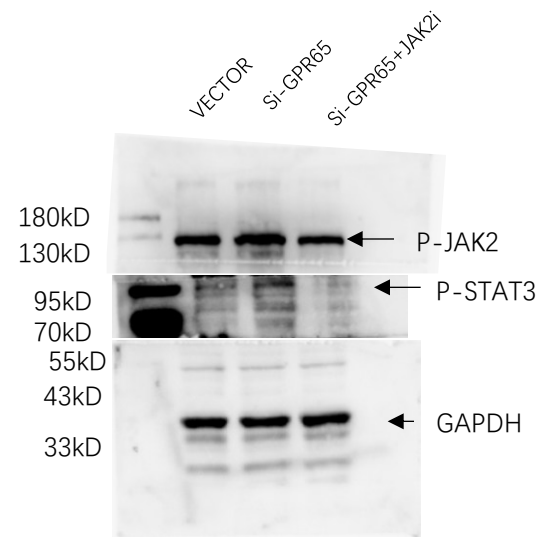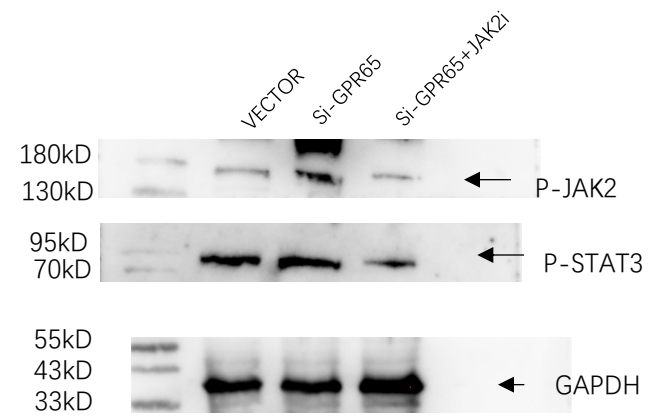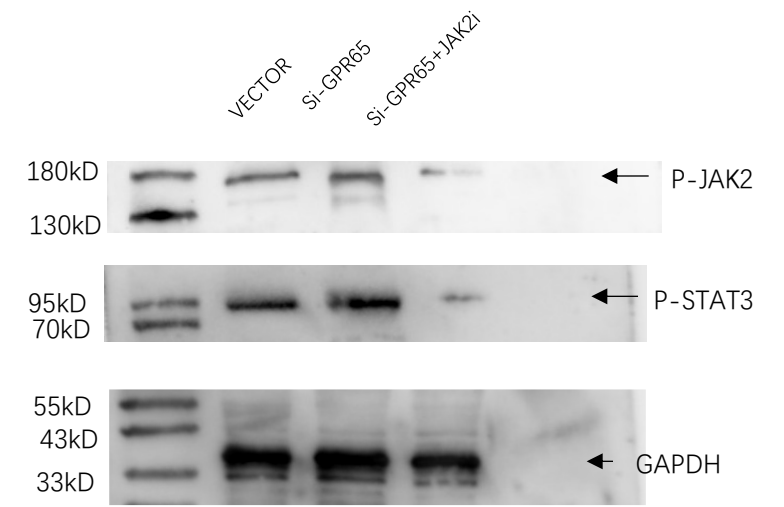

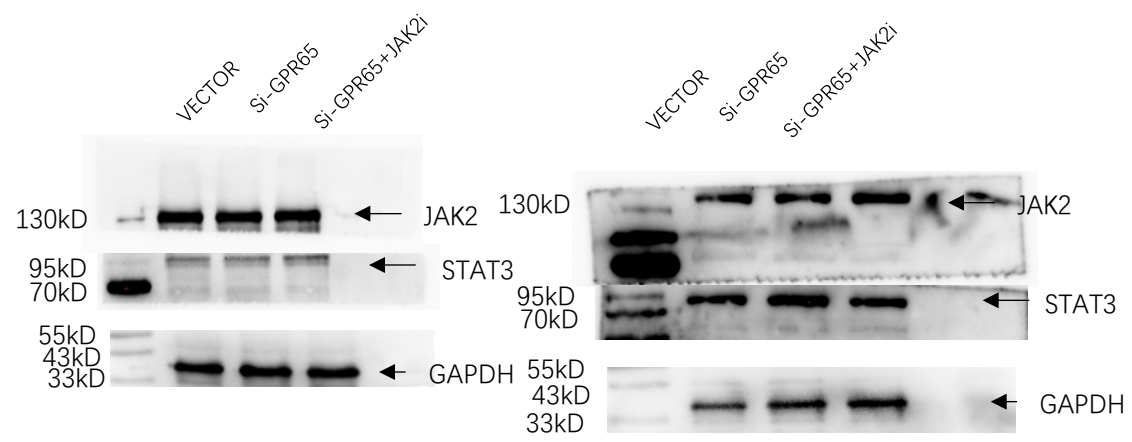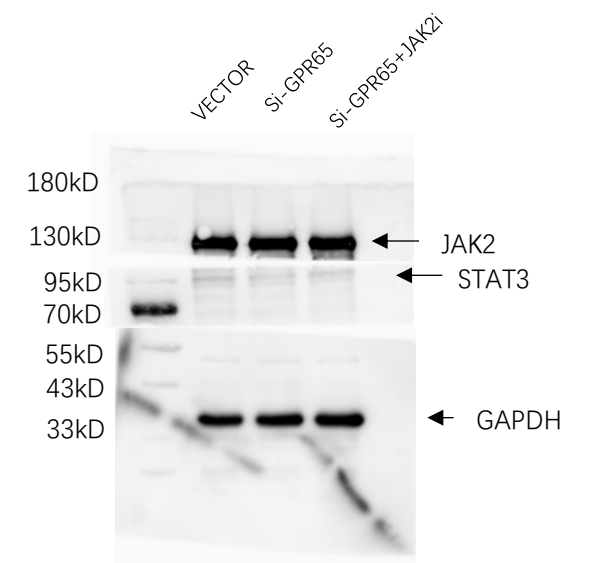

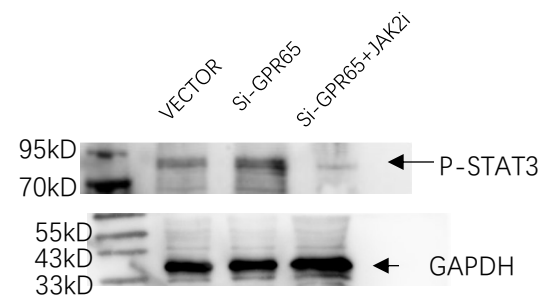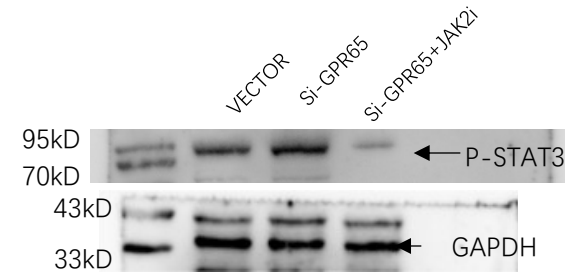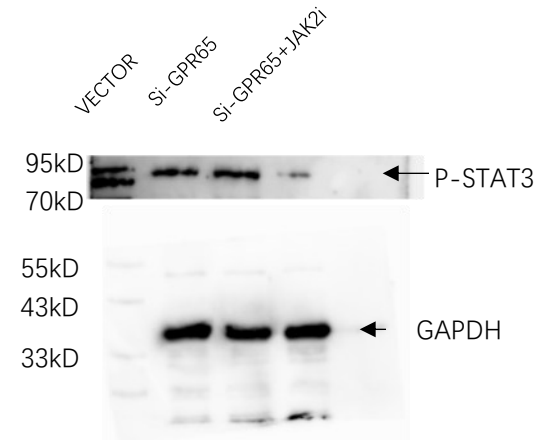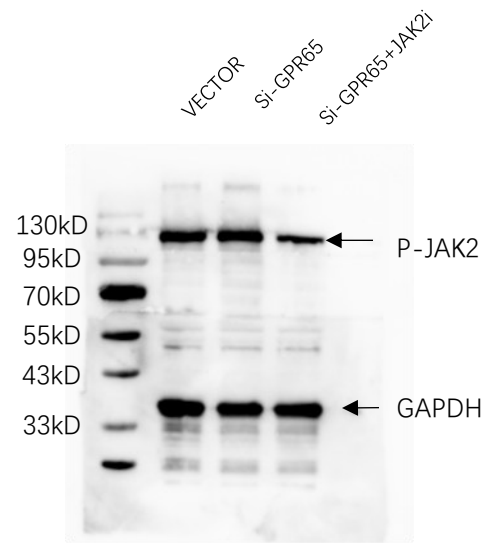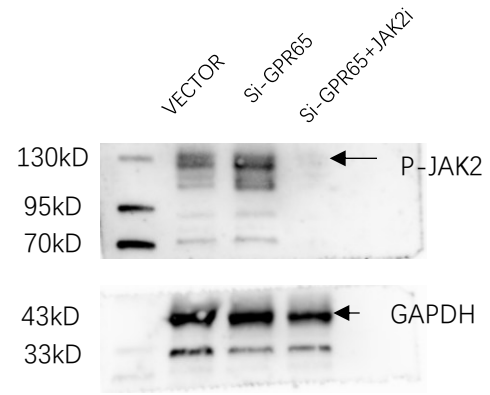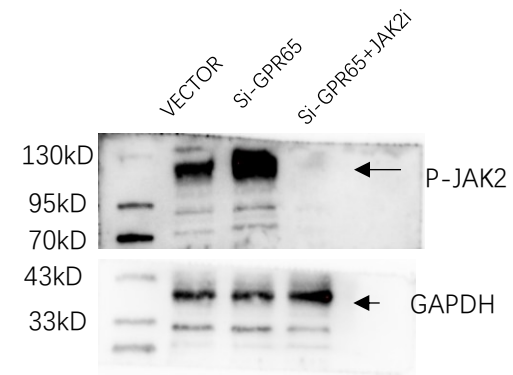

Supplement: Supplementary file 23 [file DataSheet1.pdf]

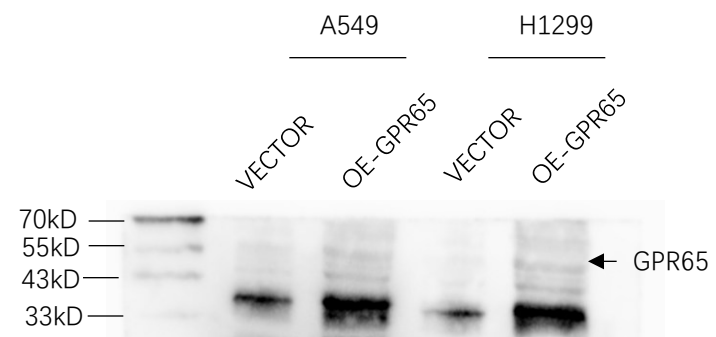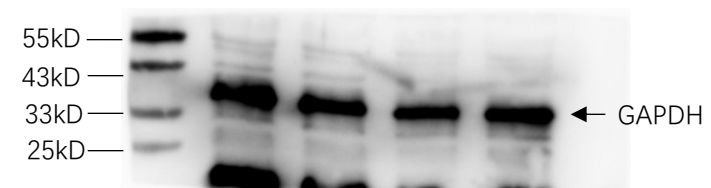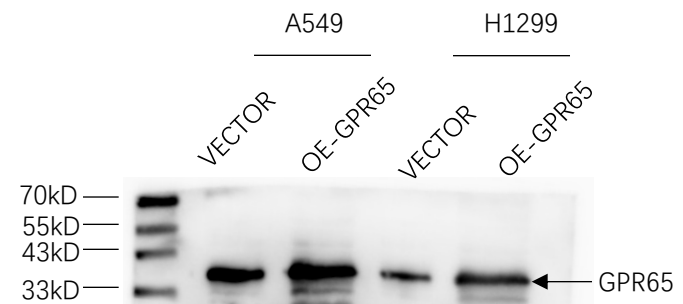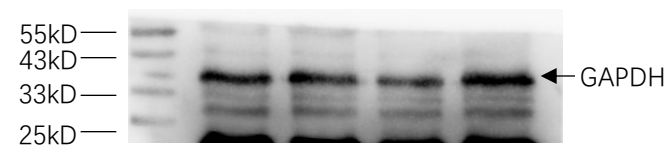

Supplement: Supplementary file 24 [file DataSheet2.pdf]

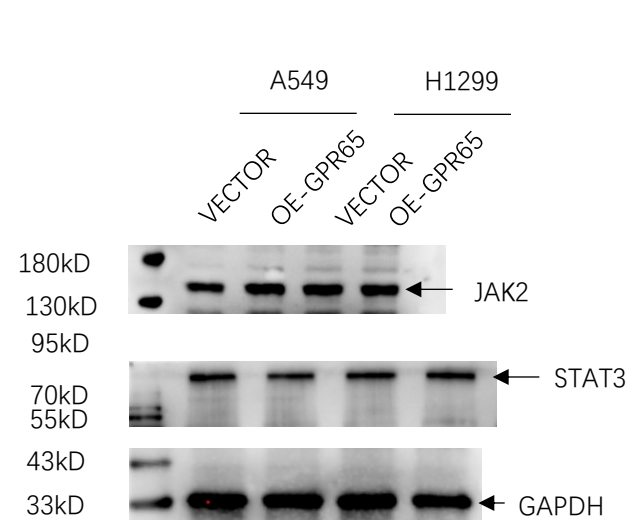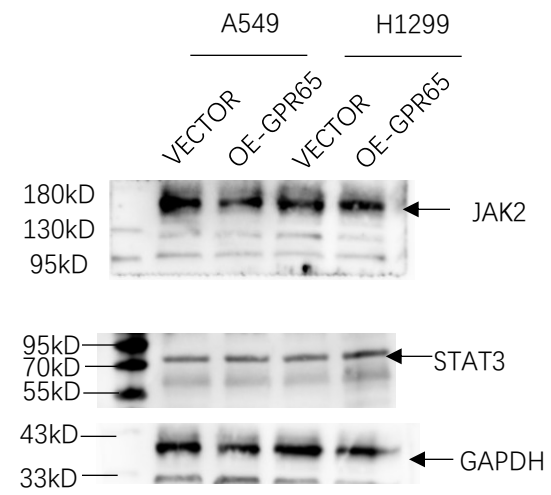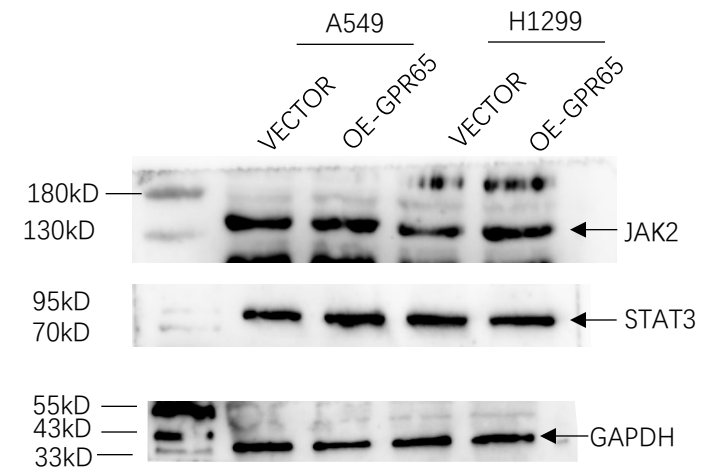

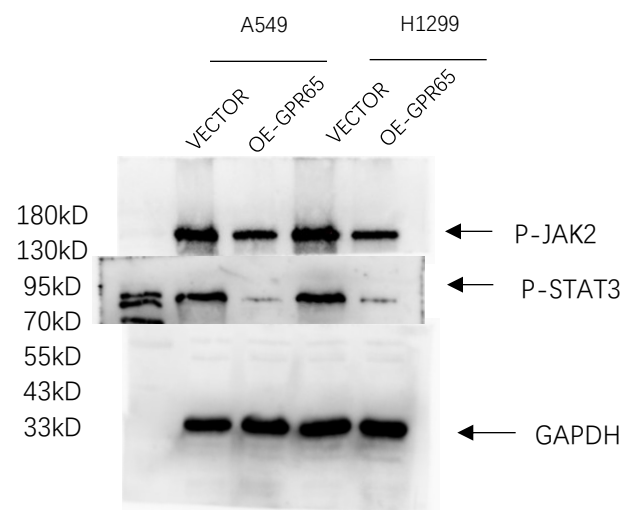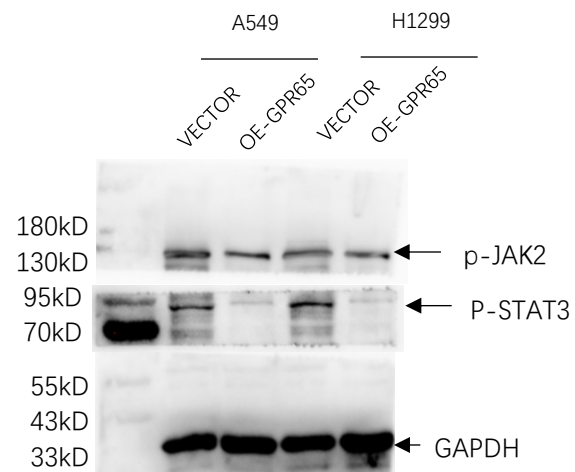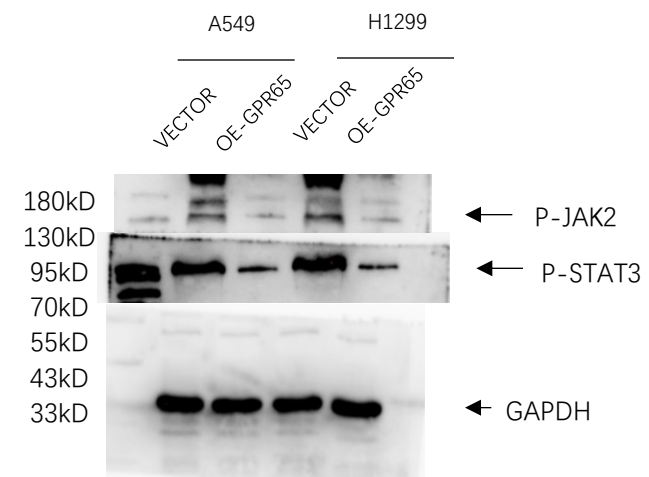

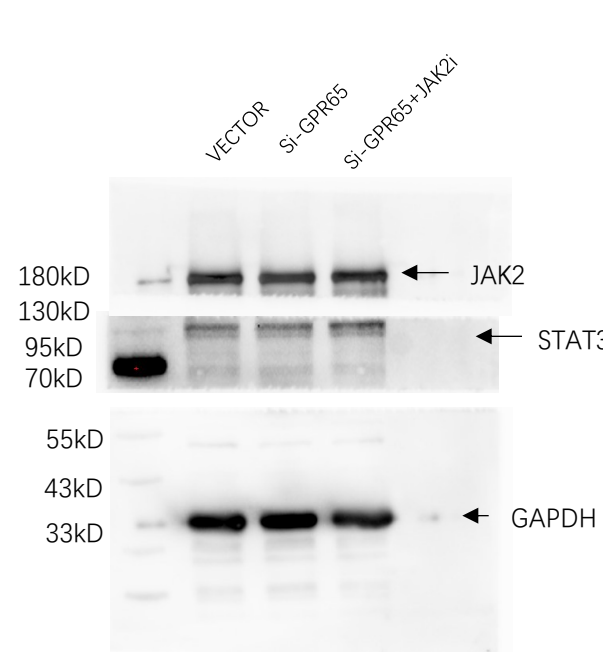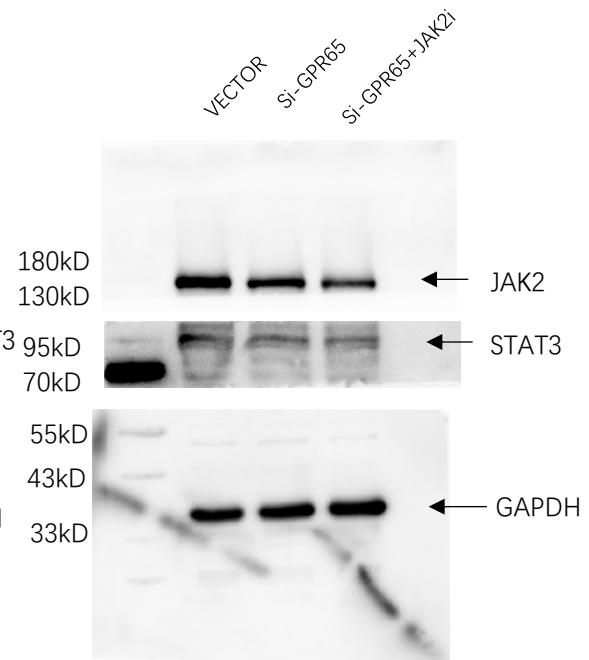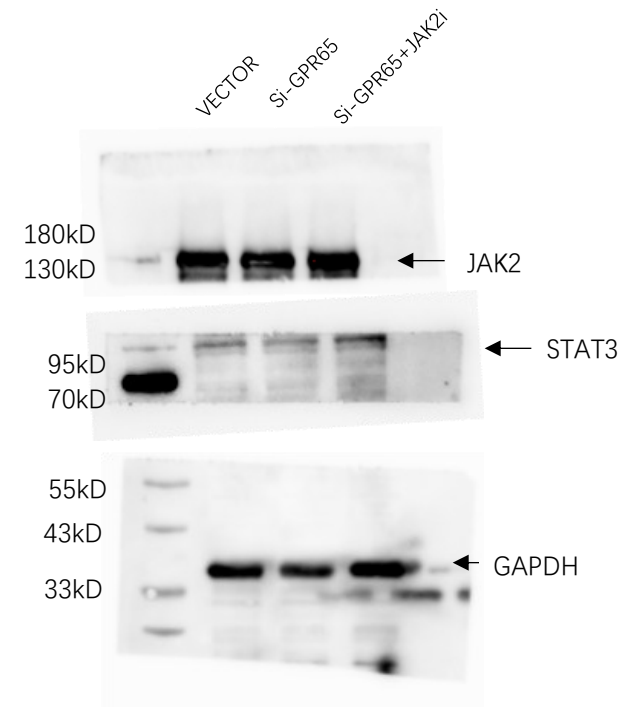

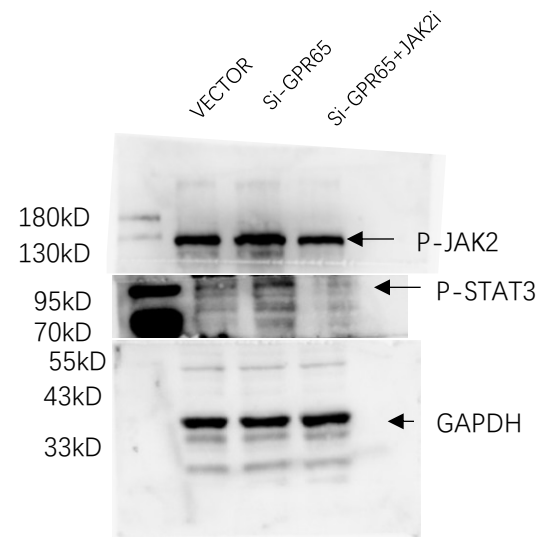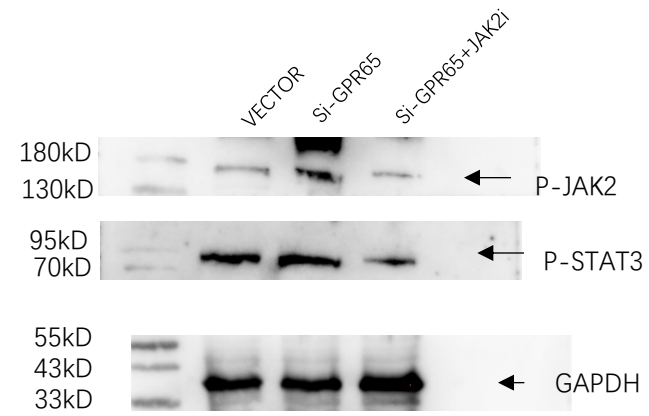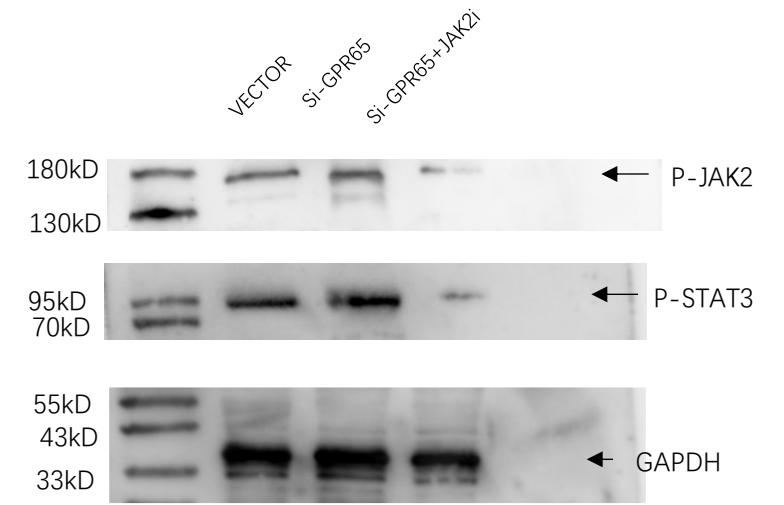

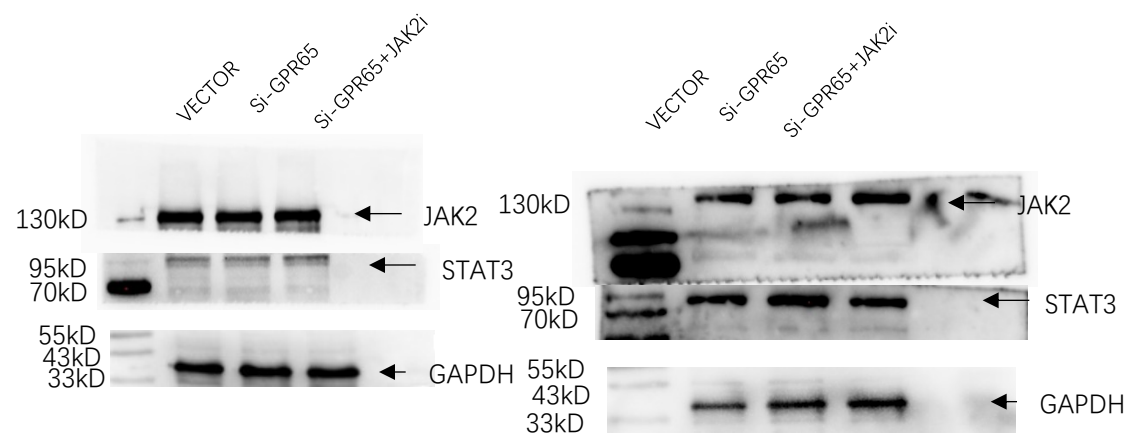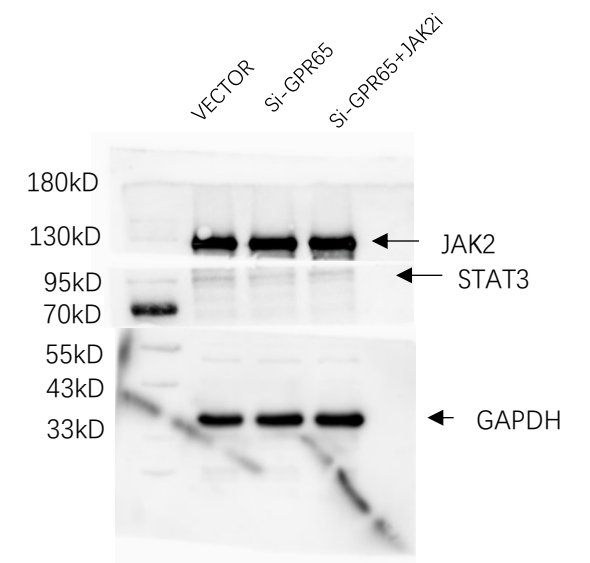

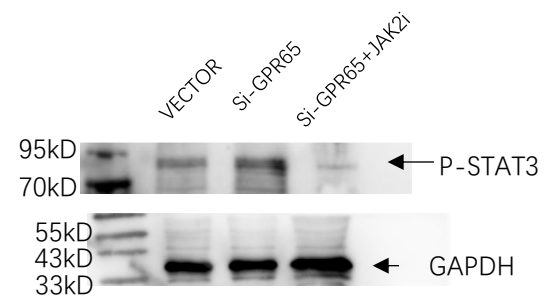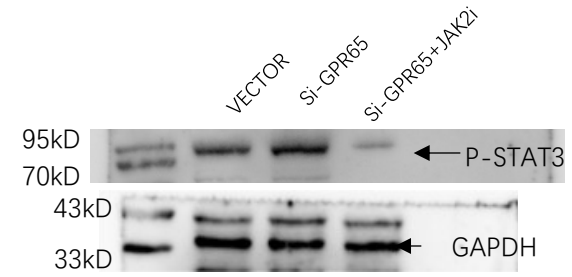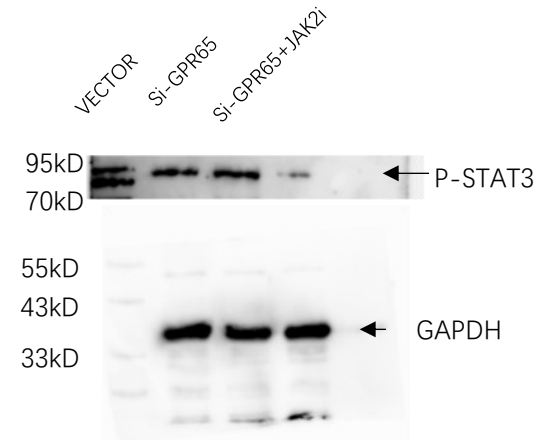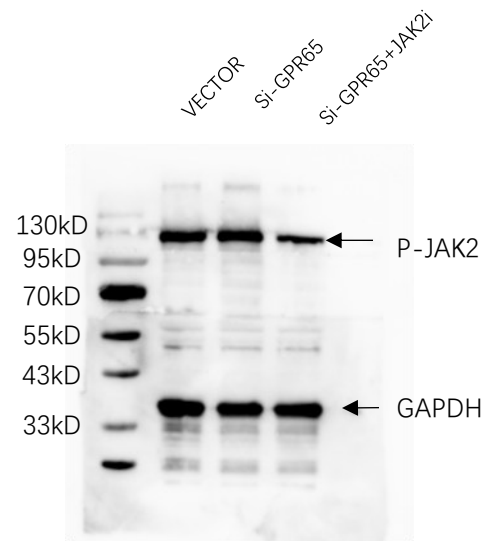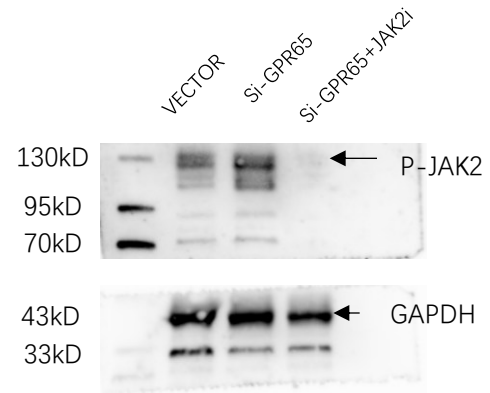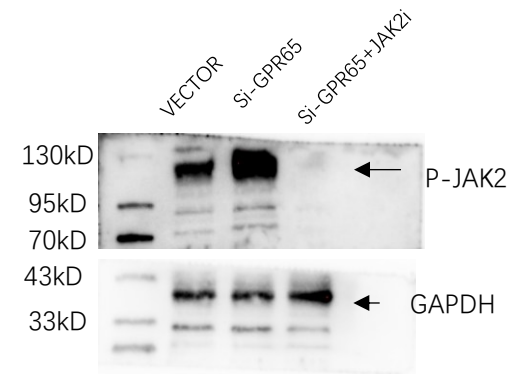

Supplement: Supplementary file 25 [file DataSheet3.pdf]

Vector

OE-GPR65

Vector

OE-GPR65

A549

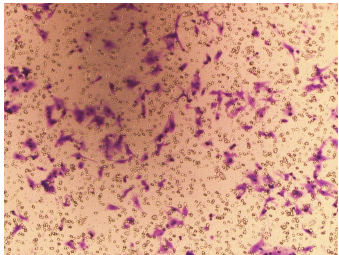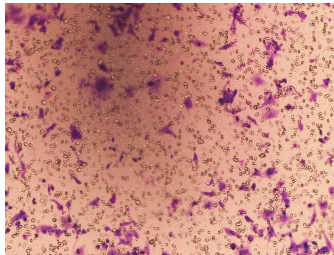

H1299

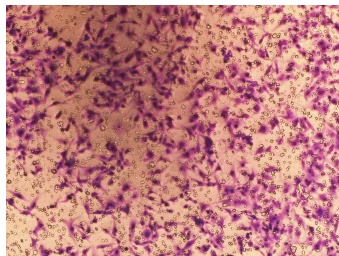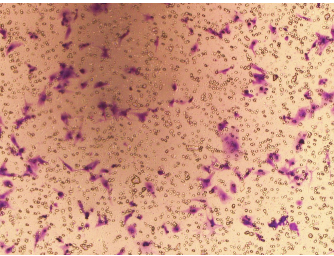

A549

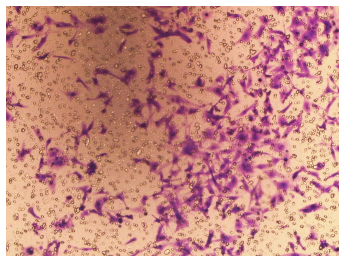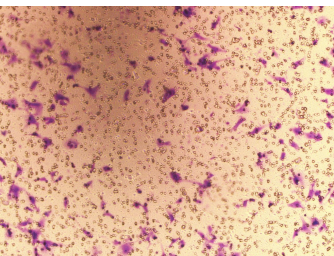

H1299

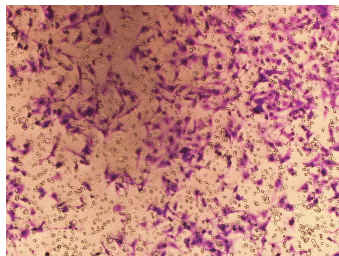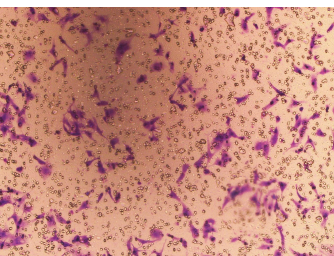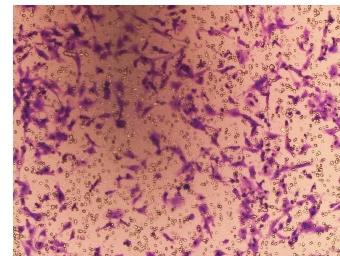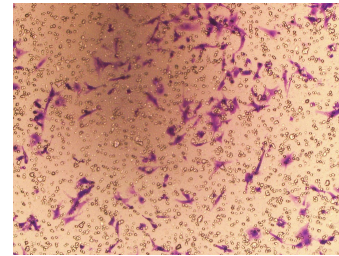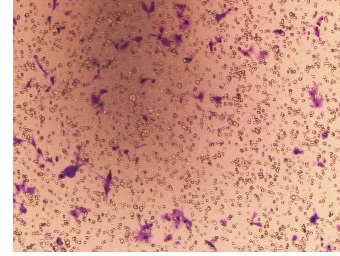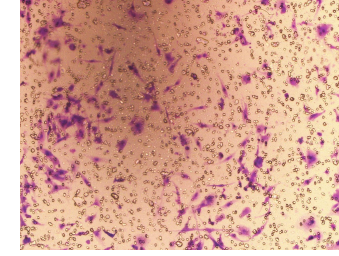

---

Migration

---

Invasion

Vector

OE-GPR65

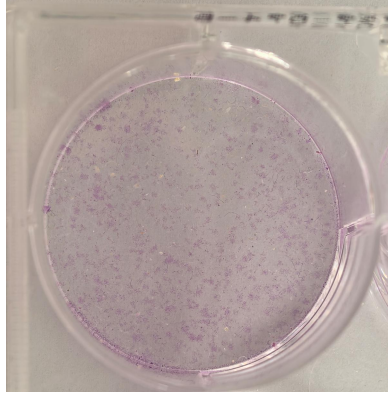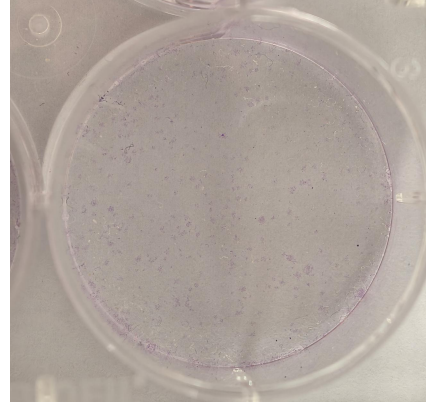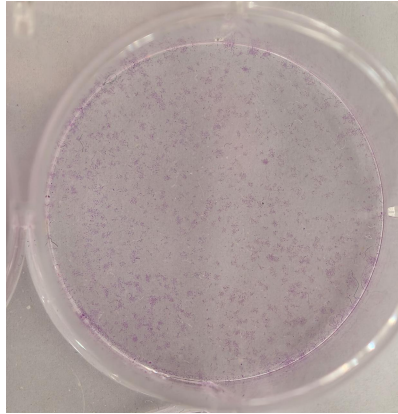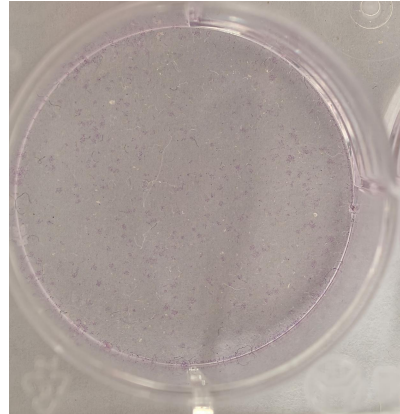

---

A549

Vector

OE-GPR65

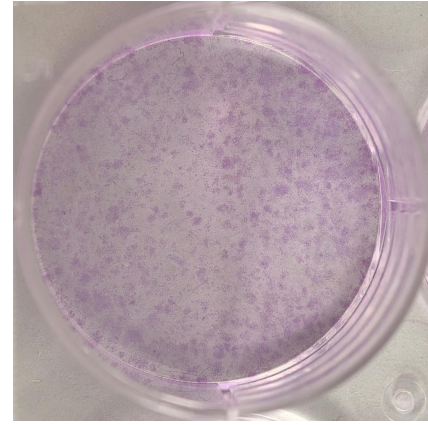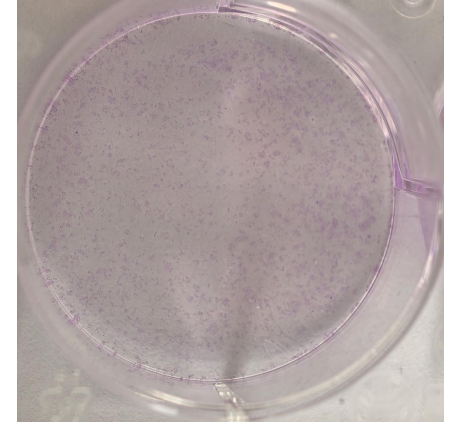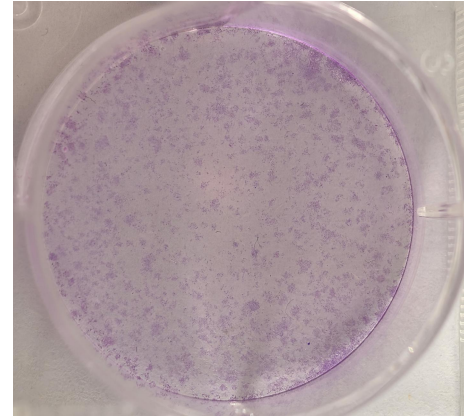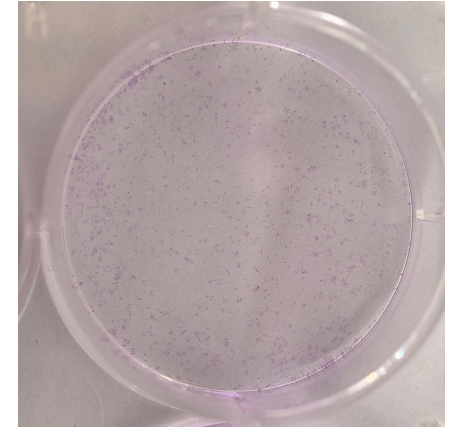

---

H1299

Supplement: Supplementary file 29 [file DataSheet7.pdf]
