# Supplementary material for: GPR65 is a novel immune biomarker and regulates the immune microenvironment in lung adenocarcinoma
Source: Front Immunol. 2025 May 30;16:1572757. doi: 10.3389/fimmu.2025.1572757 (PMC12162609; doi:10.3389/fimmu.2025.1572757)
Supplement: Supplementary file 27 [file DataSheet5.pdf]

Certificate of STR Analysis

1. **Sample**

   A549

2. **Methods**

   The sample DNA was analyzed in Genesky Biotechnologies. Inc., Shanghai.

   The PCR product was loaded into ABI 3730xl Genetic Analyzer and the STR profiling was generated by GeneMapper 5.0 software (Applied Biosystems®).

3. **Results**

Amelogenin:	X,Y
CSF1PO:	10,12
D13S317:	11
D16S539:	11,12
D5S818:	11
D7S820:	8,11
THO1:	8,9.3
TPOX:	8,11
vWA:	14

The locations of the cell sample were consistent with the STR data of **A549** cell found in the databases of ATCC, DSMZ, JCRB and EXPASY.

There was no third allele found in all the locations of the cell sample, indicating that there was no cross-contaminant of human source cell line.

Cell Bank/Stem Cell Bank

Center for Excellence in Molecular Cell Science

Chinese Academy of Sciences

Date: 2023/5/30
