# Supplementary material for: GPR65 is a novel immune biomarker and regulates the immune microenvironment in lung adenocarcinoma
Source: Front Immunol. 2025 May 30;16:1572757. doi: 10.3389/fimmu.2025.1572757 (PMC12162609; doi:10.3389/fimmu.2025.1572757)
Supplement: Supplementary file 28 [file DataSheet6.pdf]

CBTCCCAS Certificate of STR Analysis

1.Sample

NCI-H1299

2.Method and Procedure

Sample DNA was extracted by Microread Genomic DNA Kit.
PCR was amplified with STR Multi-amplification Kit(MicroreaderTM21 ID System).
PCR products were assayed with ABI 3730xl DNA Analyzer(Applied Biosystems®).
Data were analyzed using GeneMapperID-X software and then compared with the ATCC and DSMZdatabases for reference matching.

3.Results

D5S818	11
D13S317	12
D7S820	10
D16S539	12, 13
vWA	16, 17, 18
TH01	6, 9. 3
Amelogenin	X
TPOX	8
CSF1PO	12

The above results were consistent with the DNA profiles reported by ATCC, and DSMZ, and indicated no other human cell lines contamination.

Cell Bank,
Type Culture Collection,
Chinese Academy of Sciences
(CBTCCCAS)

中国科学院典型培养物保藏委员会
细胞库
